# Chemical and Pharmacological Prospection of the Ascidian *Cystodytes dellechiajei*

**DOI:** 10.3390/md22020075

**Published:** 2024-01-31

**Authors:** Pedro Jatai Batista, Genoveffa Nuzzo, Carmela Gallo, Dalila Carbone, Mario dell’Isola, Mario Affuso, Giusi Barra, Federica Albiani, Fabio Crocetta, Riccardo Virgili, Valerio Mazzella, Daniela Castiglia, Giuliana d’Ippolito, Emiliano Manzo, Angelo Fontana

**Affiliations:** 1 Bio-Organic Chemistry Unit, Institute of Biomolecular Chemistry CNR, Via Campi Flegrei 34, 80078 Naples, Italy; p.jatai@icb.cnr.it (P.J.B.); carmen.gallo@icb.cnr.it (C.G.); d.car@hotmail.it (D.C.); mario.service@libero.it (M.d.); mario.affuso@unina.it (M.A.); giusi_barra@hotmail.it (G.B.); d.castiglia@icb.cnr.it (D.C.); gdippolito@icb.cnr.it (G.d.); emanzo@icb.cnr.it (E.M.); afontana@icb.cnr.it (A.F.); 2 Department of Integrative Marine Ecology, Stazione Zoologica Anton Dohrn, 80121 Naples, Italy; fabio.crocetta@szn.it (F.C.); riccardo.virgili@szn.it (R.V.); 3 NBFC—National Biodiversity Future Center, Piazza Marina 61, 90133 Palermo, Italy; valerio.mazzella@szn.it; 4 Laboratory of Bio-Organic Chemistry and Chemical Biology, Department of Biology, University of Naples “Federico II”, Via Cupa Nuova Cinthia 21, 80126 Napoli, Italy; 5 Ischia Marine Centre, Department of Integrative Marine Ecology, Stazione Zoologica Anton Dohrn, 80077 Naples, Italy

**Keywords:** marine natural products, pattern recognition receptors (PRRs), TREM2, innate immunity

## Abstract

Marine invertebrates are a traditional source of natural products with relevant biological properties. Tunicates are soft-bodied, solitary or colonial, sessile organisms that provide compounds unique in their structure and activity. The aim of this work was to investigate the chemical composition of the ascidian *Cystodytes dellechiajei*, selected on the basis of a positive result in biological screening for ligands of relevant receptors of the innate immune system, including TLR2, TLR4, dectin-1b, and TREM2. Bioassay-guided screening of this tunicate extract yielded two known pyridoacridine alkaloids, shermilamine B (**1**) and N-deacetylshermilamine B (**2**), and a family of methyl-branched cerebrosides (**3**). Compounds **2** and **3** showed selective binding to TREM2 in a dose-dependent manner. N-deacetylshermilamine B (**2**), together with its acetylated analogue, shermilamine B (**1**), was also strongly cytotoxic against multiple myeloma cell lines. TREM2 is involved in immunomodulatory processes and neurodegenerative diseases. N-deacetylshermilamine B (**2**) is the first example of a polycyclic alkaloid to show an affinity for this receptor.

## 1. Introduction

The marine environment is a unique resource that provides bioactive natural products due to the characteristic extreme living conditions and complex biotic interactions driving the evolution of marine organisms. All these factors provide a great diversity of molecules, often with a large scope in terms of their structure and functional features [1,2]. In fact, marine organisms produce a myriad of chemical mediators and molecules involved in defense against predators and cell signaling. Many of these compounds show relevant biological activities, such as antitumor, anti-inflammatory, immunomodulatory, antiallergic, antiviral, and antiparasitic activities [3,4,5]. 

Different marine-derived drugs have reached the market as of 2023 [1]. In the last 40 years, around 29% of the approved antiviral drugs have been natural or nature-inspired compounds [6], and more than 35 marine-drug-derived pharmaceutical compounds are in clinical phases [2].

In this context, many studies have reported ascidians as sources of bioactive compounds for pharmaceutical applications due to their characteristic chemical composition. Ascidians are soft-bodied, solitary or colonial, sessile, marine organisms containing carbonate spicules that can accumulate a large range of cytotoxic compounds, such as alkaloids (especially pyridoacridines), diterpenes, sphingosines, and ceramides [5]. 

The aim of this work was to determine the chemical composition and conduct a biological evaluation of the colonial ascidian *Cystodytes dellechiajei* (Della Valle, 1877). This species is highly polymorphic in terms of its external morphology and compound production. Different chemotypes have been found in the Western Mediterranean, not always reflecting the genetic lineages recovered [7,8,9]. Among them, two genetically distinct purple morphotypes apparently produce similar pyridoacridines [7,9]. In this sense, the use of DNA barcoding is crucial for providing an accurate clade assignment and further evaluating the compound production within this species complex. 

The tunicate extract belonging to our in-house library of marine natural products has been screened within a bioassay platform to identify possible ligands of receptors involved in innate immune responses [10,11]. Particularly, we were focused on the study of immunomodulatory receptors such as human triggering receptor expressed on myeloid cells 2 (TREM2). TREM2 is emerging as an important regulator of immunological responses [12,13], and it has also been implicated in the modulation of microglial activity and survival. TREM2 mutations have been linked to neurodegenerative disorders such as Alzheimer’s disease and multiple sclerosis [14,15]. Moreover, TREM2 plays an important role in other pathophysiological inflammatory processes. 

TREM2 seems to be a promiscuous receptor, and its specific natural ligands remain unknow so far [16,17,18]. Recently, we reported sulfavant A (SULF A), a nature-inspired sulfolipid with promising adjuvant activity, as the first synthetic small molecule able to bind to TREM2 [19]. 

**Figure 1:**
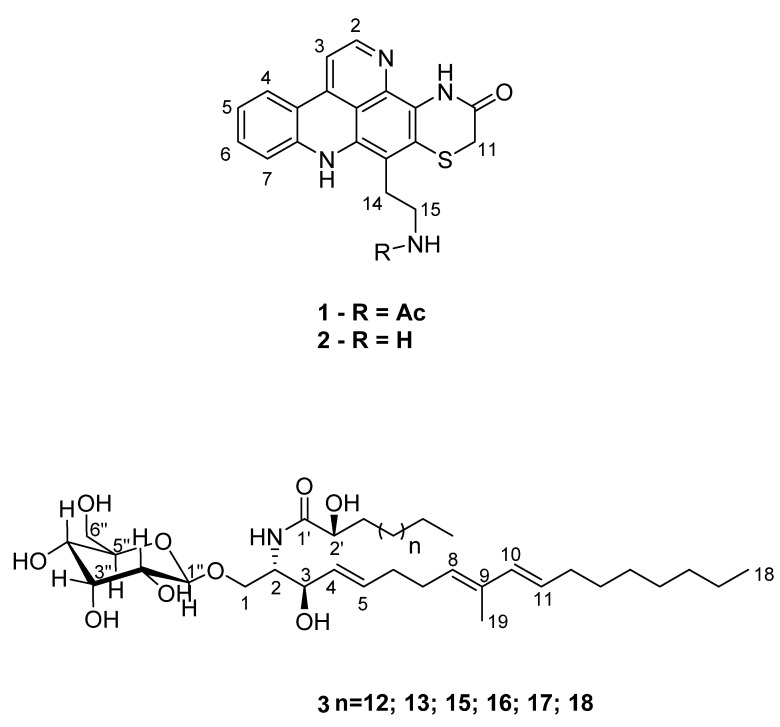
Compounds isolated and identified from the extract of *C. dellechiajei*.

Here, we report a chemical investigation of *C. dellechiajei* collected in Cartaromana Bay (Ischia Island, Italy), whose enriched extract obtained using solid-phase extraction (SPE) was active in TREM-receptor-binding assays. The fractionation of the active extract resulted in the identification of two known pyridoacridine alkaloids, shermilamine B (**1**) and N-deacetylshermilamine B (**2**), and a family of cerebroside-like lipids (**3**) (Figure 1). The structural identification of the bioactive molecules was carried out by using nuclear magnetic resonance (NMR) and mass spectrometry (MS) techniques.

## 2. Results

### 2.1. Identification of the Colonies

**Figure 2:**
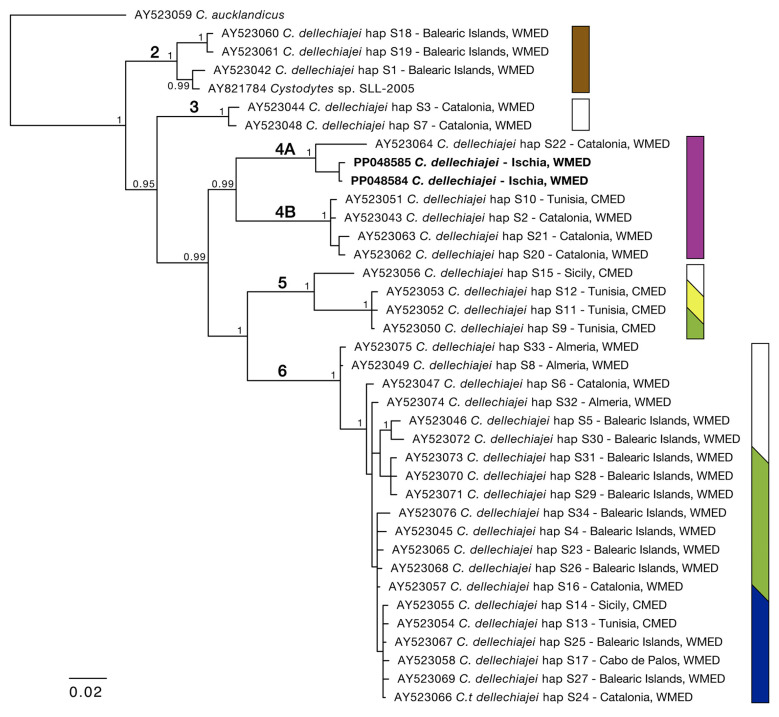
Bayesian phylogenetic tree of the genus *Cystodytes* based on the COI alignment (545 bp). Numbers in bold on the branches follow reference [7]. Posterior probabilities > 0.90 are reported at nodes. Samples examined in this study are reported in bold. The colored bars represent the external coloration of the colonies in each clade, as reported in [7,8].

The purple colonies were morphologically identified as *C. dellechiajei* due to the presence of the flat discoidal spicules typical of this species. The two COI sequences (545 bp; GenBank accession number: PP0485845) showed a 96.2–96.3% similarity with a purple-morph *C. dellechiajei* haplotype (AY523064) from Catalonia, Spain [7], and lower similarities (87.2–90.1%) with another 31 sequences attributed to the same nominal species and mostly originating from the Mediterranean. The BI phylogenetic tree was based on the COI multiple sequence alignment (MSA) made from 38 sequences of 545 bp. The BI tree (Figure 2) placed our samples within clade 4 sensu [7], which includes purple-morph specimens from the Western and Central Mediterranean with shermilamine B as the main pyridoacridine [7]. Despite sharing their external coloration and principal compound, this clade actually forms two distinct subclades with a 9.1–10.5% dissimilarity between them. For the sake of clarity, we hereby labelled these clades 4A and 4B, with our samples from Ischia falling within clade 4A together with the specimen from Catalonia (AY523064) (Figure 2). 

### 2.2. Screening Platform and Bioactive Molecule Isolation

In agreement with our previous work [20,21], an aliquot (60 mg) of a *C. dellechiajei* extract was fractionated and screened in a bioassay platform based on solid-phase extraction using chromabond HR-X columns.

**Figure 3:**
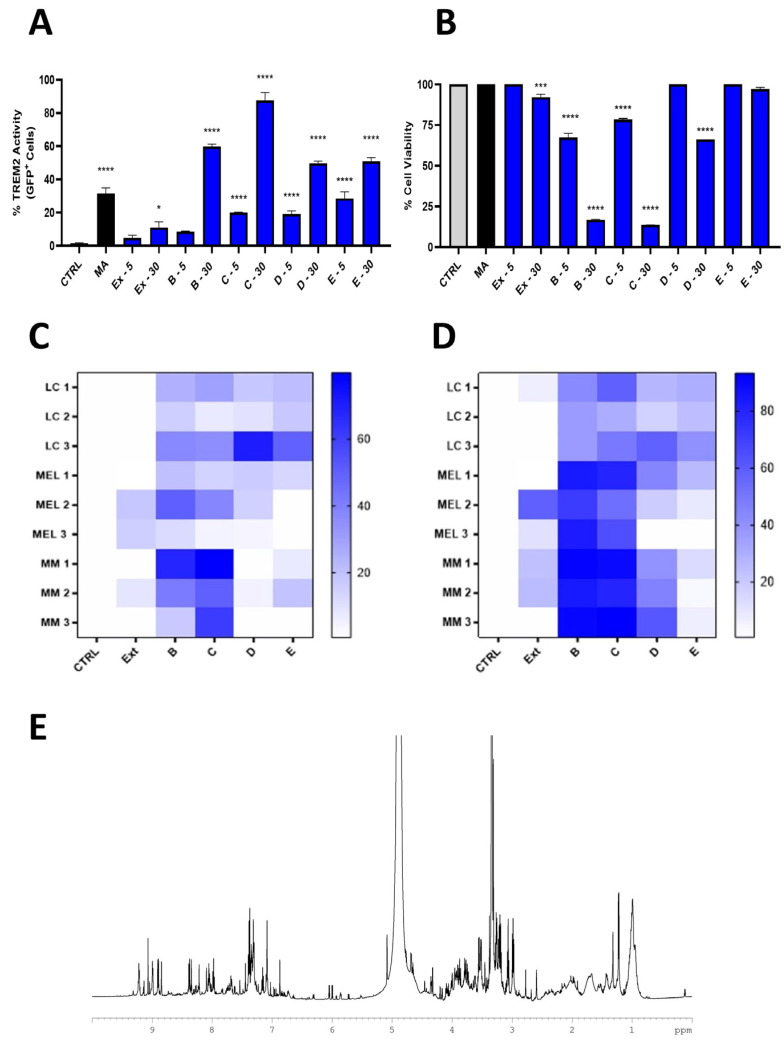
Data obtained through screening on our in-house platform. (**A**) TREM2-reporter-cell activity of the extract and HRX fractions (**B**–**E**) at concentrations of 5 and 30 μg/mL; CTRL = cells treated only with vehicle (MeOH); MA = mycolic acid used as positive control at 10 µg/mL. (**B**) Cell viability analysis on TREM2 reporter cells; statistical analysis was performed using two-way ANOVA. (**C**,**D**) Heat maps of viability assays of the *C. dellechiajei* extract and related HRX fractions (**B**–**E**) carried out on a panel of nine different cell lines (the blue color means percentage of cell growth inhibition): LC 1 (CALU-1), LC 2 (HCC827), LC 3 (CALU-3), MEL 1 (A2058), MEL 2 (A375), MEL 3 (MALME-3M), MM 1 (JJN-3), MM 2 (KMS-12), and MM 3 (RPMI 8226). Graph reports the viability values of the cells treated with 5 (**A**) and 30 (**B**) µg/mL of total extract (EX) and HRX-SPE fractions (**B**–**E**). Values reported in the color bar legend on the right indicate the % percentage of vitality (white = 100% vitality; dark blue = 0% vitality). Cells with 1% DMSO in 0.1 mL of medium were used as blank. (**E**) ^1^H NMR spectrum of fraction B in MeOD, 600 MHz. * *p* < 0.05; *** *p* < 0.001, **** *p* < 0.0001.

To select for immune-response-enhancing molecules against neoplastic cells, the fractions were tested at two concentrations of 5 μg/mL and 30 μg/mL on a panel of reporter cell lines for relevant innate immune system receptors, including TLR2, TLR4, dectin-1b, and TREM2. The initiation of innate immunity was given by the binding of active molecules to a pattern recognition receptor (PRR). Toll-like receptors (TLRs) are one of the best-characterized families of PRRs, and they play a critical role in host defenses against infection. However, this platform also considered tests on dectin-1b and TREM2 as representative members of new classes of receptors with immunomodulatory activity. Positive results were obtained only for the TREM2 cell lines (Figure 3 and Figure S1). After incubation, the receptor activation was measured as the percentage of GFP+ cells using flow cytometry, in comparison to mycolic acid, which is one of the suggested bona fide ligands [22]. Fractions B and C at 30 μM showed the best activity on the TREM2 receptor, despite a reduction in cell viability below the value of 25%. 

The fractions were also tested on a panel of cancer cells, including lung cancer (LC), melanoma (MEL), and multiple myeloma (MM) cell lines. Fractions B and C showed low cytotoxic activity except for their effect against the myeloma cell lines (JJN3, KMS-12, and RPMI), with these lines showing a higher sensitivity (Figure 3C,D).

A chemical analysis of active fraction B indicated the presence of a mixture of medium–high-polarity compounds (Figure 3E). The proton NMR spectrum in MeOD at 600 MHz (Bruker spectrometer) showed characteristic down-shifted signals of the aromatic protons between 9.0 and 7.0 ppm; a clear doublet at 6.1 ppm, which is characteristic of an α-β unsaturated system; and several signals between 4.3 and 2.9 ppm for the presence of methine linked to electronegative heteroatoms, such as oxygen. The spectrum of fraction C was similar to that of fraction B, with the enrichment of signals in the region of oxymethine and aliphatic protons (see Figure S2). 

### 2.3. Isolation and Structure Characterization of the Bioactive Molecules

Starting from the TLC distribution of the products in active fractions B and C, the extract was fractionated on silica gel, followed by purification on RP-SPE or silica columns.

Three major compounds were isolated on the basis of the correlation of the ^1^H NMR signals with fraction B. We assigned the presence of the aromatic signals between 8.5 and 7.0 ppm to two pyridoacridine alkaloids belonging to the shermilamine family (compounds **1** and **2**). On the other hand, the signal at 6.01 ppm was identified as the proton at C-10 in an 8,10-diene system of a methyl-branched cerebroside (compound **3**). The sugar moiety of this product also accounted for the presence of the numerous oxymethyne protons observed in active fractions B and C. 

Shermilamine B (**1**) was obtained as an orange resin. The HRESIMS analysis showed peaks at 391.1209 [M + H]^+^ and *m*/*z* 389.1074 [M − H]^−^ that were in agreement with the molecular formulae C_21_H_19_N_4_O_2_S (calcd. for *m*/*z* 391.1223) and C_21_H_17_N_4_O_2_S (calcd. for *m*/*z* 389,1078), respectively (Figure S4). The ^1^H NMR spectrum (Figure S3) showed the diagnostic signals for pyridoacridine alkaloids (δ_H_ 8.55 (H-2, d *J* = 5.1 Hz), 7.50 (H-3, d *J* = 5.1 Hz), 8.02 (H-4, d *J* = 8.2 Hz), 7.11 (H-5, m), 7.46 (H-6, overlapped), and 7.45 (H-7, overlapped)), together with three methylene groups (δ_H_ 3.55 (H_2_-11, s), 3.33 (H_2_-14, overlapped), and 3.14 (H_2_-15, br t)) and one methyl (δ_H_ 1.92 (s H_3_-18)). The assignment (Table 1) was fully confirmed through a comparison with the literature data [4,23].

N-deacetylshermilamine B (**2**) was obtained as a red resin. The molecular formula was deduced as C_19_H_16_N_4_OS through an analysis of HRESIMS (*m*/*z* 349.1127 [M + H]^+^, calcd. for C_19_H_17_N_4_OS *m*/*z* 349.1118, and *m*/*z* 347.0961 [M − H]^−^, calcd. for C_19_H_15_N_4_OS *m*/*z* 349.0972) (Figure S6). Compared with compound **1**, the difference of 42 *amu* in the mass spectra suggested the loss of an acetyl moiety. In addition, the major fragment observed in the MS/MS spectra was the peak at *m*/*z* 332.0868, indicating a terminal amino alkyl group. The ^1^H NMR spectrum (Table 1, Figure S5) was virtually identical to that of compound **1** and displayed the same six diagnostic signals for pyridoacridine alkaloids (δ_H_ 8.54 (H-2, d *J* = 5.0 Hz), 7.49 (H-3, d *J* = 5.0 Hz), 8.00 (H-4, d *J* = 8.1 Hz), 7.09 (H-5, t *J* = 7.8 Hz), 7.44 (H-6, t *J* = 7.8 Hz), and 7.53 (H-7, d *J* = 8.1 Hz)), as well as the three methylene groups (δ_H_ 3.63 (H_2_-11, s), 3.33 (H_2_-14, t *J* = 8.0 Hz), and 3.14 (H_2_-15.1, t *J* = 8.0 Hz)). NMR spectra were also acquired in DMSO-d_6_ (Table 1), and the data were in good agreement with the NMR assignment reported for compound **2** in the literature [4,24].

**Figure 4:**
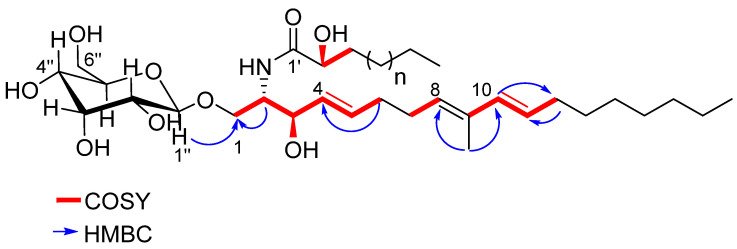
Key ^1^H-^1^H COSY (red line) and HMBC (blue arrows) correlations of compound **3**.

Compound **3** was obtained as a white resin. The NMR data (Figure S12) revealed the presence of five different signals in the olefinic region: δ_H_ 6.01 (H-10, d *J* = 15,8 Hz)/δ_C_ 136.0, 5.58 (H-11, dt *J* = 6.8; 15.5 Hz)/δ_C_ 128.6, 5.38 (H-8, t *J* = 7.2; 14.5 Hz)/δ_C_ 130.3, 5.77 (H-5, dt *J* = 6.4; 15.4 Hz)/δ_C_ 134.3, 5.53 (H-4, dd *J* = 7.5; 15.2 Hz)/δ_C_ 131.3. The ^1^H – ^1^H COSY correlation (Figure S8), associated with the long-range correlation of a methyl singlet at δ_H_ 1.74/δ_C_ 12.0 with the carbons at δ_C_ 130.3 and 136.0 (Figure S13), suggested the presence of an 8,10-diene system, as reported for glucoceramides isolated from organisms belonging to different phyla [25,26,27,28,29]. The key long-range correlations between H-6 and C-4, H-10 and C-12, and H-12 and C-11 helped to locate the double bonds in the structure (Figure 4). The geometry of the double bond between C-4 and C-5 and the diene moiety were assigned as *E* by their large vicinal coupling constants (Table 2). The comparison of the chemical shifts and coupling constant of protons H-10 and H-11 of **3** in the literature [26,27,28] indicates an *s-trans* stereochemistry for the diene system. An α-glucopyranose moiety was observed in the chemical shifts of ^1^H and ^13^C (between δ_H_ 4.29 and δ_H_ 3.70 for ^1^H, and δ_C_ 104.8 and δ_C_ 62.7 for ^13^C) (Table 2). The analysis of the HSQC spectra (Figure S9) showed the connectivity between the anomeric proton H-1″ δ_H_ 4.29 (d *J* = 7.82 Hz) and the carbon C-1″ δ_C_ 104.8. The HMBC correlation between H-1″ and C-1 (Figure 4) confirmed that the glucose unit was attached to C-1. It is worthwhile to note that most of the monoglycosylceramides reported in the literature have a sugar unit in the β-orientation. Through the analysis of the chemical shift and the coupling constant of an anomeric proton (δ_H_ 4.29 (d, *J* = 7.82 Hz)/δ_C_ 104.8), it was possible to establish that compound **3** also had a sugar unit in the β-orientation; this value is indicative of an axial–axial relationship [25,26]. The presence of a signal in the ^1^H NMR spectra at δ_H_ 4.02 linked to the carbon δ_C_ 54.6, along with the ^1^H–^1^H COSY correlations between H-1 and either H-2 or H-3, confirmed the 2-amino-1,3-dioxygenated moiety, whereas the hydrogen H-2’ at δ_H_ 4.02, linked to the carbon at δ_C_ 73.1 in the HSQC spectra, showed a correlation in the ^1^H–^1^H COSY spectra with H-3′ at δ_H_ 1.57, which is characteristic of the 2′-hydroxy fatty acid system. Once again, the comparison of chemical shifts and coupling constants of H-1, H-2, and H-2′ (Table 2) with the data in the literature was consistent for the configuration *2S,3R,2′R* (*erythro*) [25,26,27,28].

The LC-MS/MS data (Figure S17) showed a series of deprotonated peaks with 14 *amu* of difference ([M − H]^−^ = 738.55; 752.57; 780.60; 794.62; 808.63; and 822.65), indicating a complex mixture of homologous compounds. These data are in accordance with the formula C_31_H_55_NO_9_ + *n*CH_2_ (*n* = 12 to 18). The MS/MS (Figures S18–S23) data analysis showed the loss of the sugar unit in the negative mode (*m*/*z* 179.05) and the common loss of 162 *amu* [M − H − glucose]^-^ and 180 *amu* [M − H − glucose − H_2_O]^−^. On the basis of biosynthetic consideration and previous reports, these data suggested that the long-chain base corresponded to 2-amino-1,3-dihydroxy-9-methyl-4,8,10-octadecatriene, thus leading to compound **3** being assigned as a mixture of α-glucopyranose-9-methyl-cerebrosides, differing only in the chain length of the α-hydroxy fatty acid moiety linked to the amino function of the branched sphingosine [25,26,27,28].

### 2.4. Biological Activity

**Figure 5:**
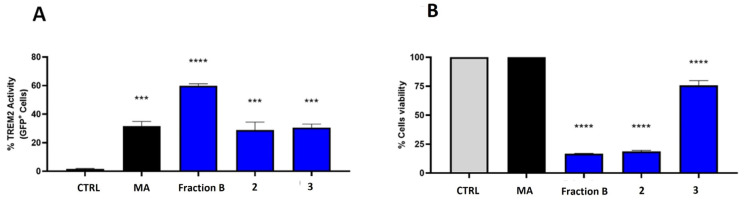
(**A**) The 2B4 human-TREM2/DAP12 reporter cells stimulated with compounds at 30 μg/mL, compared to HR-X B fraction. Receptor activity (GFP expression) was evaluated after overnight incubation using flow cytometry. (**B**) Effect of the compounds on cell viability; CTRL = cells treated only with vehicle (MeOH); MA = mycolic acid used as positive control at 10 µg/mL. Statistical analysis was performed using two-way ANOVA. *** *p* < 0.001, **** *p* < 0.0001.

To validate the identification, compounds **1**–**3** were tested on TREM2 reporter cells and the results were compared with the response to fraction B, obtained from the screening on HR-X SPE. The receptor activity of the HRX-B fraction was stronger than that of the single products, but compounds **2** and **3** showed a biological profile that was significantly similar (Figure 5A). Indeed, cerebroside mixture **3** showed a good affinity for the receptor, with an increase of up to 30% in the number of GFP^+^ cells at 30 μg/mL (Figure 5B). The mixture also showed a low toxicity with a cell viability higher than 75% at 30 μg/mL. Compound **2** showed the same binding activity, but also revealed a remarkable toxicity near 80% at the same concentration. Notably, the binding activity of **2** and **3** with TREM2 was comparable to that achieved with mycolic acid (MA), which has recently been reported to be specifically recognized by the receptor in a mechanism of host immunity control induced by mycobacteria. The effect of compound **1** on the TREM2 activity was not evaluable, since the fraction was very toxic, even at concentrations lower than 30 μg/mL.

**Figure 6:**
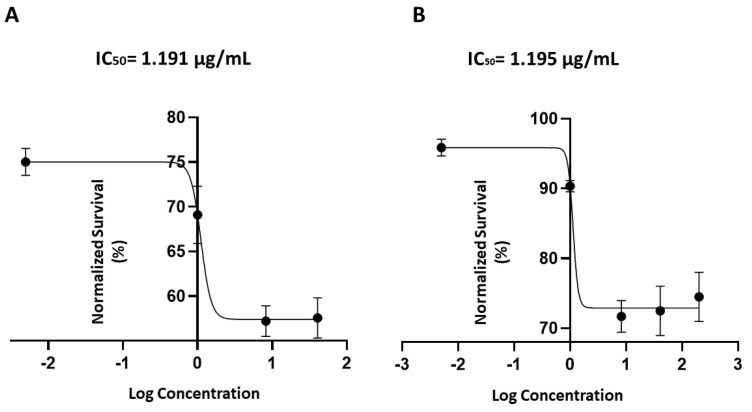
Dose–response curve for compound (**2**) on percentage of cell viability after treating JJN3 (**A**) and KMS12 (**B**) myeloma multiple cells with a range of concentrations, from 0.1 to 10 µg/mL. A nonlinear regression analysis was performed for the estimation of the IC_50_ value, as plotted in the figure.

Compound **2** was also evaluated for its cytotoxic activity on the myeloma cancer cell lines JJN-3 and KMS-12. This compound showed the same biological activity on both cell lines, with an IC_50_ value of about 1.2 μg/mL (3.4 μM) (Figure 6).

## 3. Discussion

In this work, an extract of *C. dellechiajei* was assessed using our bioassay platform, which is designed for the discovery of small molecules with immunomodulatory activity [22]. In particular, we found that at least two metabolites present in the extract of the tunicate, namely *N*-deacetylshermilamine B (**2**) and the mixture of glucosylceramides (**3**), can bind to the regulatory receptor TREM2.

TREM2 belongs to a family of receptors occurring on the membrane of innate immune cells [30]. Recently, we reported sulfavant A as the first synthetic compound able to specifically bind to TREM2 [19]. Sulfavant A is derived from sulfoquinovosides in the diatom *Thalassioisra weissfloidgii* [19]. Together with the deglycosylated mycolic acid (MA), other natural compounds have been suggested as TREM2 ligands, including anionic and zwitterionic lipids, nucleic acids, lipoprotein particles, phospholipids, and heat shock proteins [31,32]. However, to date, a physiological ligand for this receptor is still unknown and the data provided to support the activity of these compounds are only indirect. 

As reported in Figure 4, fraction **3** rich in glucosylceramides showed the best result on the TREM2 receptor assay. It is worth noting that compound **3** differed from the other sphingolipids isolated from tunicates due to the presence of a 4,8,10-triene system. This is the first report of those compounds in these invertebrates. This family of glucosylceramides was previous isolated from the starfishes *Cosmasterias lurida*, *Allostichaster capensis* (formerly *A. inaequalis*), and *Ophidiaster ophidianus* [27,28,29], and other analogous cerebrosides with the same 4,8,10-triene system have been isolated from the octocoral *Sarcophyton ehrenbergi* [26]. It is known that this class of lipid plays a significant role in the mammal immune system and in the angiogenic process [32]. Also, these lipids can show cytotoxic, antitumor, antifungal, antimicrobial, and antiviral biological activities, among others [33]. Sarcoehrenoside A and B, for instance, were both able, in an anti-inflammatory assay, to reduce the iNOS protein expression that is up-regulated in LPS-stimulated cells [26]. The biological activity of compound **3** suggests the possibility of using these molecules to stimulate the innate immune system, with potential applications in cancer immunotherapy. 

Compounds **1** and **2** were significantly cytotoxic when tested on innate immune cells. However, to the best of our knowledge, this is the first report of the cytotoxicity of N-deacetylshermilamine B (**2**) against the multiple myeloma cell lines JJN-3 and KMS-12. Previous studies have reported cytotoxic activities for various shermilamine types, such as shermilamine B (**1**), shermilamine D, and shermilamines F and C, on human oral, colon, melanoma, and lymphoma cancer cell lines with IC_50_ values between 0.5 μM and 10.0 μM [34,35,36]. The IC_50_ value (3.4 μM) of compound **2** for the myeloid cancer cell lines was in accordance with the data reported in the literature for this class of natural products on other cancer cell lines.

Shermilamine B, cystodins A-I, and kuanoniamine D are common metabolites in the green, blue, and purple morphs of *C. dellechiajei* [5,23]. For the purple morph, the chemotype is based on the sulfur-containing pyridoacridines and their *N*-deacetylated forms [37]. This class of compound carries out several biological activities, notedly antiviral, antimicrobial, immunosuppressant, antiparasitic, anticholinesterase, cytotoxic, and insecticidal activities [4,5,23].

In addition, the cytotoxicity of different pyridoacridine alkaloids, such as kuanoniamine A, *N*-deacetylkuanoniamine, cystodytins A and C (both isolated from *C. dellechiajei*), sagitol C, 12-deoxyascididemin, and ascididemin, have been evaluated on a large range of cancer cell lines (namely colon, lymphoma, leukemia, and lung cancers, among others), with IC_50_ values between 0.2 and 7.63 μM [38,39,40,41,42].

## 4. Materials and Methods

### 4.1. General Experimental Procedures

The NMR spectra were recorded on a Bruker DRX 600 spectrometer (600 MHz for ^1^H, 150 MHz for ^13^C) equipped with a three-channel inverse (TCI) CryoProbe. Chemical shift values were reported in ppm (δ) and referenced to the internal signals of residual protons (C_6_D_6_ ^1^H δ 7.15, ^13^C 128.0 ppm; CDCl_3_ ^1^H δ 7.26, ^13^C 77.0 ppm; and MeOD ^1^H δ 3.34, ^13^C 49.0). High-resolution mass spectra were acquired on a Q-Exactive Hybrid Quadrupole–Orbitrap Mass Spectrometer (Thermo Scientific, Milan, Italy). All the chemicals and solvents (Sigma Aldrich, St. Louis, MO, USA) were of analytical reagent grade and were used without any further purification.

### 4.2. Sample Collection and Molecular Identification 

Several colonies of the purple morph of *C. dellechiajei* were collected by handpicking them in Cartaromana Bay (Ischia Island, Gulf of Naples, ~40.7236 N, 13.9603 E) at up to a 1 m depth. The samples were kept in plastic bags with filtered seawater and transported alive to the Benthos Laboratory of the Stazione Zoologica Anton Dohrn of Naples (SZN). The colonies were then cleaned of external debris and contaminants and frozen at −20 °C. A sub-sample of two different colonies was isolated, examined under a stereomicroscope to confirm the morphological identification, and fixed in 99% ethanol for subsequent molecular analyses. The samples were deposited in the collection of the Laboratory of Benthos (SZN_B_2518ASC32F, SZN_B_2519ASC32G). To assess the genetic lineage of the Ischia population, the total genomic DNA was extracted from the zooids using the DNeasy Blood and Tissue kit (QIAGEN, Valencia, CA, USA), as described in [43]. A partial region of the *cytochrome c oxidase subunit I* (COI) mitochondrial gene was amplified via PCR using the primers and conditions described in [43]. The obtained amplicons were purified and sequenced as described in [43]. The chromatograms obtained were checked for quality and assembled with Unipro UGene v.39 (v.39, Unipro, Novosibirsk, Russia) [44]. The cleaned sequences were then compared with the available reference sequences in the NCBI nucleotide database through BLASTn (www.ncbi.nih.gov, accessed on 22 December 2023). All the available COI sequences of *Cystodytes* present in the database were downloaded, aligned, and used to build a Bayesian inference (BI) tree to assess the phylogenetic position of our samples. The BI tree was built with MrBayes (v.3.2.5) [45], and the best model was chosen with the iqtree (v2.1.2) model selection option [46]. The Markov chain Monte Carlo (MCMC) method was run for 10 million generations, and this was sampled every 1000 generations with summarized parameters and a burn-in of 25%. The run convergence was checked with Tracer (v1.7.1, BEAST Developers) [47]. 

### 4.3. Extraction and Isolation of Metabolites 

The tunicate samples were lyophilized to yield 44 g of purple powder (dry weight). About 10 g of dry biomass was extracted with methanol (Merk Life Science S.r.l., Milan, Italy) using a tissue homogenizer, Precellys Evolution, equipped with a cooling system, Cryolys Evolution (Bertin Italia, Genoa, Italy), to obtain 580 mg of crude extracts. This protocol of extraction consisted of a run at 6200 rpm (3 cycles × 30 s) with a temperature of 16 °C to prevent degradation, followed by the centrifugation of the sample at 3450 rpm for 10 min at 4 °C. The extract was filtered with a rinsed filter paper and dried in a rotatory evaporator using a maximum temperature of 24 °C. This full crude extract was kept at −80 °C until further use.

A small amount of raw extract was fractionated on 6.0 mL/500 mg of HR-X resin following the protocol reported in our previous work to obtain five enriched extracts, labeled from A to E [20]. Both the raw extract and the HRX fractions were screened in the bioassay platform. Fractions B and C were the most active fractions, while fractions D and E were weakly active. Fractions B and C, eluted with MeOH/water in a 50:50 ratio and ACN/water in a 7:3 ratio, respectively, were selected for further study.

The distribution of metabolites in the enriched SPE fractions was analyzed using thin-layer chromatography (TLC) and ^1^H NMR. 

Through careful observation of the TLC analysis, it was possible to select the main spots of fraction B in the crude extract. Thus, the raw extract (270 mg) was submitted to chromatographic fractionation on a SiO_2_ column with a gradient of eluents as follows: petroleum ether/diethyl ether, 9:1 (30 mL) or 8:2 (50 mL); chloroform/methanol, 99:1 (40 mL), 9:1 (50 mL), or 7:3 (30 mL); and chloroform/methanol/water, 65:25:4 (30 mL). The procedure resulted in 29 new fractions. The fraction eluted with 99:1 chloroform/methanol (less than 0.1 mg) was an orange resin identified as the known pyridoacridine alkaloid shermilamine B (**1**). The fraction obtained with 7:3 chloroform/methanol (2.0 mg) was further fractionated using cartridge C18-hydra following a gradient of MeOH in water before an elution step with 1:1 chloroform/MeOH. This last fraction contained a red resin, identified as the known pyridoacridine alkaloid N-deacetylshermilamine B (**2**) (0.21 mg, 0.078%). The silica fraction eluted with 9:1 chloroform/MeOH (0.30 mg) was further purified using a Pasteur pipette with SiO_2_ resin following a gradient of MeOH in chloroform, to obtain the fractions enriched in glycosphingolipids (**3**, 0.1 mg, 0.037%).

All the isolated compounds were analyzed using NMR and ESI-MS (Figures S3–S16). Compound **3** was further analyzed using LC-MS-MS/MS at 50 μg/mL (Figure S17) according to the methods previously reported by our group for the analysis of lipids [48] on a biphenyl column (Kinetex, 2.6 μm, 150 × 2.1 mm), using 60/40 MeOH/H_2_O (pH of 8; NH_4_OH 0.005M) as the initial mobile phase. The method of analysis followed the following gradient: 60/40 to 80/20 in 2 min. Then, 100% MeOH was reached in 15 min and was held in an isocratic mode of 100% MeOH for 15 min. The flow used was 0.3 mL/min and the volume of injection was 20 μL.

Shermilamine B (**1**): ^1^H NMR (MeOD, 600 MHz), see Table 1. *m*/*z* 391,1209 [M + H]^+^, calcd. for C_21_H_19_N_4_O_2_S *m*/*z* 391,1223; *m*/*z* 389,1074 [M − H]^−^, calcd. for C_21_H_17_N_4_O_2_S *m*/*z* 389,1078.

N-deacetylshermilamine B (**2**): ^1^H NMR (MeOD, 600 MHz; DMSO-d_6_, 600 MHz), see Table 1. *m*/*z* 349,1127 [M + H]^+^, calcd. for C_21_H_19_N_4_O_2_S *m*/*z* 349,1118; *m*/*z* 347,0961 [M − H]^−^, calcd. for C_21_H_17_N_4_O_2_S *m*/*z* 347,0972.

Compound **3**: ^1^H NMR (MeOD, 600 MHz), see Table 2. LC-MS-MSMS analysis, see Figures S17–S23.

### 4.4. TREM2 Reporter Assay

The 2B4 mouse T cell hybridomas, stably transfected with enhanced green fluorescent protein (eGFP) under consensus sequences of NFAT and human TREM2/DAP12 cDNA and then selected using G418 (1 mg/mL), were kindly provided by prof. Marco Colonna from Washington University in St. Louis. The cells were maintained in an RPMI 1640 medium (GIBCO) supplemented with 10% fetal bovine serum (GIBCO) and cultured for up to 25 passages (max. confluency, 1 × 10^6^ cells/mL). The compounds were dissolved in MeOH and administered on the plate by coating them using 0.05 mL of the solution of the molecules at the indicated concentration. Once the solvent was evaporated, 1.5 × 10^5^ cells were plated onto flat 96-well plates in 0.2 mL of medium and incubated overnight at 37 °C and 5% of CO_2_. After incubation, the reporter cells were analyzed using a MACSQuant^®^ Analyzer 16 Flow Cytometer (Milteny Biotec, Biotec, Auburn, CA, USA) in terms of the GFP^+^ cell percentage. Mycolic acid (Sigma-Aldrich, CAS number: 37281-34-8) was used at 10 ug/mL (30% GFP^+^ cells) as a positive control [22]. The cell viability was assayed by staining for 5 min in the dark with propidium iodide (Invitrogen™, catalog number: P3566) at 500 ng/mL.

### 4.5. Cytotoxicity Assay

The human cell lines A2058, CALU-1, CALU-3, HCC827, MALME-3 M, and A375 were purchased from the American Type Culture Collection (ATCC); 3 multiple myeloma lines, KMS-12, RPMI 8226, and JJN-3, were purchased from the German Collection of Microorganisms and Cell Cultures (DSMZ). All the cells were cultured as previously reported by [17]. Each cell line was tested in the cytotoxic assay at a concentration of 1 × 10^4^ in 0.1 mL of medium, in a 96-well plate. The organic fractions were diluted to a maximum concentration of 3 mg/mL in DMSO and tested at 5 and 30 μg/mL. Cells with 1% DMSO in 0.1 mL of the medium were used as the blank. Cisplatin, MEK inhibitor, and doxorubicin were all used as positive controls at a concentration of 100 μM. All the conditions were plated in duplicate, and the cells were incubated for 24 h. For an evaluation of the IC_50_ of the pure compounds, the assays were performed in triplicate. For the cell lines growing in adherence, the Sulforodamine B (SRB) Assay Kit (Abcam ab235935, Milan, Italy) was used. After 24 h of treatment, the cells were fixed and stained according to the manufacturer’s instructions. The optical density was determined at 565 nm. An MTS Proliferation Assay Kit (Abcam, ab197010, Milan, Italy) was used for cells growing in suspension. An amount of 10 μL of MTS (3-(4,5-dimethylthiazol-2-yl)-5-(3-carboxymethoxyphenyl)-2-(4-sulfophenyl)-2H-tetrazolium) was added to each well, and the plates were incubated at 37 °C for 4 h. The absorbance was measured at 490 nm. For all the experiments, the percentage of cytotoxicity was calculated as: ((O.D. vehicle) × (O.D. sample)/O.D. vehicle) × 100. A background correction was carried out by subtracting the O.D. of the culture media.

### 4.6. Binding Activity of TLR2, TLR4, and Dectin-1b

Human TLR-4/NF-kB/SEAP, TLR2/NF-kB/SEAP, and dectin-1b/SEAP HEK 293 reporter cells (Invivogen, San Diego, CA, USA) were plated in flat-bottomed 96-well plates at 2 × 10^4^ (TLR2 and TLR4) and 2 × 10^5^ (dectin-1b) cells/well and stimulated for 16 h with fractions and positive controls: 10 ng/mL of Pam3CSK4 (InvivoGen) for TLR2 and 1 μg/mL of LPS (Santa Cruz Biotechnology™, Dallas, TX, USA) for TLR4 and zymosan (InvivoGen) at a concentration of 100 μg/mL in the HEK-Blue™ detection medium. The receptor-activation-induced substrate hydrolysis by secreted alkaline phosphatase (SEAP) was measured at 640 nm according to the manufacturer’s instructions.

## 5. Conclusions

We selected an extract of the ascidian *C. dellechiajei* collected in Ischia Island, Italy, and determined its activity in binding to TREM2, a surface receptor involved in the activation and control of the response of several cells of the innate immune system. The chemical investigation of the extract led us to isolate and identify the known pyridoacridine alkaloid *N*-deacetylshermilamine B (**2**) and a family of methyl-branched sphingolipids as potential ligands of the immune receptor. *N*-deacetylshermilamine B (**2**) is the first alkaloid to show the ability to bind to TREM2. Along with its analogue shermilamine B (**1**), **2** showed selective cytotoxicity against the myeloid cancer cell lines JJN-3 and KMS-12. 

Despite the presence in the sample of signals indicating some impurities, which could have influenced the results, the combination of the immunomodulatory and cytotoxic activity of *N*-deacetylshermilamine B (**2**) may suggest the potential development of this product as an inducer of immunogenic cell death. Furthermore, it is worth noting that crude fraction B obtained from preliminary SPE processing showed stronger activity than the pure isolated products. Fraction B was composed of a mixture of compounds **1**–**3** together with minor uncharacterized constituents. It is, therefore, possible that chemical analogues **2** or **3** with more potent activity could also be present in the *C. dellechiajei* extract. Furthermore, a cooperative mechanism of the mixture in reinforcing the immunomodulatory properties cannot be excluded.

## Figures and Tables

**Table 1:** NMR data of **1** and **2** (600 MHz).

	1	2					
No.	δ_H_^a^ (mult, *J* in Hz)	δ_H_^a^ (mult, *J* in Hz)	*δ*_C_^a^	δ_H_^b^ (mult, *J* in Hz)	*δ*_C_^b^	*δ*_H_^d^ (mult, *J* in Hz)	*δ*_C_^d^
**2**	8.55 (d, 5.1)	8.54 (d, 5.0)	151.5	8.55 (d, 5.5)	150.8	8.55 (d, 5.3)	150.5
**3**	7.50 (d, 5.1)	7.49 (d, 5.0)	108.8	7.56 (d,5.2)	107.7	7.63 (d, 5.3)	107.3
**3a**			141.8		140.3		142.5
**3b**			117.4		115.5		115.4
**4**	8.02 (d, 8.2)	8.00 (d, 8.1)	125.1	8.05 (d, 8.1)	124.1	8.14 (d, 8.1)	125.0
**5**	7.11 (m)	7.09 (t, 7.8)	123.0	7.04 (d, 7.9)	121.3	7.13 (d, 7.9)	122.2
**6**	7.46 (overlapped)	7.44 (t, 7.8)	133.3	7.43 (d, 7.9)	131.5	7.53 (t, 7.9)	132.7
**7**	7.45 (overlapped)	7.53 (d, 8.1)	118.0	7.73 (d, 8.1)	116.7	7.48 (d, 8.1)	116.9
**7a**			141.4		139.8		140.0
**8a**			133.6		131.9		131.5
**9**			107.3		107.8		108.4
**9a**			123.5		124.0		124.4
**11**	3.55 (s)	3.63 (s)	30.5	3.61 (s)	29.1	3.64 (s)	29.4
**12**			166.5		163.8		164.0
**13a**			123.3		121.9		121.7
**13b**			139.4		137.2		136.0
**13c**			118.6		116.9		117.1
**14**	3.33 (overlapped)	3.33 (t, 8)	28.1	3.24 (t, 7.5)	27.1 ^c^	3.21 (t, 8.1)	25.8
**15**	3.14 (bt)	3.14 (t, 8)	38.7	2.91 (t, 7.5)	37.3 ^c^	2.98 (brt)	36.9
**18**	1.92 (s)	-				-	

^a^ solvent CD_3_OD; ^b^ solvent DMSO-d_6_; ^c^ not detected in the 13C spectrum; assigned aided by HSQC experiment; ^d^ Bontemps, N. et al. *J. Nat. Prod*. **2010**, *73*, 1044–1048 [24].

**Table 2:** ^1^H and ^13^C (600 and 150 MHz in MeOD) NMR data of **3**.

3		
No.	δ_H_ (mult, *J* in Hz)	*δ_C_*
**1**	3.73 (dd, 3.6; 10.3) 4.14 (dd, 4.7; 10.6)	69.7
**2**	4.02 (m)	54.6
**3**	4.17 (bt, 7.4)	73.1
**4**	5.53 (dd, 7.5; 15.2)	131.3
**5**	5.77 (dt, 6.4; 15.4)	134.3
**6**	2.12 (m)	34.0
**7**	2.24 (m)	30.4
**8**	5.38 (t, 7.2)	130.3
**10**	6.01 (d, 15.8)	136.0
**11**	5.58 (dt, 6.8; 15.5)	128.6
**12**	2.11 (m)	33.5
**13–17**	1.37–1.30	23.8–31.0
**18**	0.94–0.91	14.5
**19**	1.74 (s)	12.0
**2′**	4.02 (m)	73.1
**3′**	1.57 (m)	36.0
**(CH_2_)*_n_***	1.37–1.30	23.8–31.0
**CH_3_**	0.94–0.91	14.5
**1″**	4.29 (d, 7.82)	104.8
**2″**	3.23 (m)	75.0
**3″**	3.38 (m)	77.9
**4″**	3.30 (m)	71.6
**5″**	3.31 (m)	78.0
**6″**	3.90 (d, 11.9) 3.70 (dd, 4.12; 11.2)	62.7

## Data Availability

The data are contained within the article and/or are available from the corresponding author upon reasonable request.

## Supplementary Materials

The following supporting information can be downloaded at: https://www.mdpi.com/article/10.3390/md22020075/s1, Figure S1: Evaluation of the toll-like receptor (TLR) and dectin-1b activation with 5 and 30 µg/mL of total extract (ext) and HRX-SPE fractions (B–E) in TLR-2 (**A**), TLR-4 (**B**), and dectin; (**C**) NF-kB/SEAP reporter cell lines. Pam2CSK4 (PAM) and LPS were used as positive controls for TLR-2 and TLR-4, respectively, while zymosan was used as positive control for dectin assay. Asterisks indicate significant differences compared to the cells treated only with vehicle (control, Ctrl) at a 95% (*P*  <  0.05) confidence level, as determined using two-way ANOVA; Figure S2: ^1^H NMR spectrum of bioactive fraction B (600 MHz, MeOD); Figure S3: ^1^H NMR spectrum of compound **1** (600 MHz, MeOD); Figure S4: Mass spectra (ESI—positive and negative modes) of compound **1**; Figure S5: ^1^H NMR spectrum of compound **2** (600 MHz, MeOD); Figure S6: HSQC NMR spectrum of compound **2** (600 MHz, MeOD); Figure S7: ^13^C NMR spectrum of compound **2** (600 MHz, MeOD); Figure S8: ^1^H NMR spectrum of compound **2** (600 MHz, DMSO-d_6_); Figure S9: HSQC NMR spectrum of compound **2** (600 MHz, DMSO-d_6_); Figure S10: ^13^C NMR spectrum of compound **2** (600 MHz, DMSO-d_6_); Figure S11: Mass spectra (ESI—positive and negative modes) of compound **2**; Figure S12: ^1^H NMR spectrum of fraction containing compound **3** (600 MHz, MeOD); Figure S13: ^1^H-^1^H COSY spectra of fraction containing compound **3** (400 MHz, MeOD); Figure S14: HSCQ NMR spectra of fraction containing compound **3** (600 MHz, MeOD); Figure S15: HMBC NMR spectra of fraction containing compound **3** (600 MHz, MeOD); Figure S16: ^13^C NMR spectra of fraction containing compound **3** (600 MHz, MeOD); Figure S17: LC-MS/MS spectrum (ESI—negative mode) of fraction containing compound **3**; Figure S18: MS/MS spectrum (ESI—negative mode) of the main peak at *m*/*z* 738.55; Figure S19: MS/MS spectrum (ESI—negative mode) of the main peak at *m*/*z* 752.57; Figure S20: MS/MS spectrum (ESI—negative mode) of the main peak at *m*/*z* 780.60; Figure S21: MS/MS spectrum (ESI—negative mode) of the main peak at *m*/*z* 794.61; Figure S22: MS/MS spectrum (ESI—negative mode) of the main peak at *m*/*z* 808.63; Figure S23: MS/MS spectrum (ESI—negative mode) of the main peak at *m*/*z* 822.65.
