# Supplementary material for: Chemical and Pharmacological Prospection of the Ascidian Cystodytes dellechiajei
Source: Mar Drugs. 2024 Jan 31;22(2):75. doi: 10.3390/md22020075 (PMC10890457; doi:10.3390/md22020075)

Chemical and Pharmacological Prospection of the Ascidian *Cystodytes dellechiajei*

Pedro Batista[1], Genoveffa Nuzzo[1*], Carmela Gallo[1], Dalila Carbone[1], Mario dell'isola[1], Mario Affuso[1], Giusi Barra[1], Fabio Crocetta[2], Riccardo Virgili[2,5], Valerio Mazzella[3,4], Daniela Castiglia[1], Giuliana d'Ippolito[1], Emiliano Manzo[1], Angelo Fontana[1,5]

1. Bio-Organic Chemistry Unit, Institute of Biomolecular Chemistry CNR, Via Campi Flegrei 34, Pozzuoli, 80078 Naples, Italy
2. Department of Integrative Marine Ecology, Stazione Zoologica Anton Dohrn, 80121 Naples, Italy
3 Department of Integrative Marine Ecology, Stazione Zoologica Anton Dohrn, Ischia Marine Centre, Ischia 80077, Naples, Italy
4 NBFC, National Biodiversity Future Center, Piazza Marina 61, 90133 Palermo, Italy
5 Laboratory of Bio-Organic Chemistry and Chemical Biology, Department of Biology, University of Naples "Federico II", Via Cupa Nuova Cinthia 21, Napoli 80126, Italy
* Correspondence: nuzzo.genoveffa@icb.cnr.it

Figure S1. Evaluation of the Toll-Like Receptor (TLR) and Dectin 1b activation with 5 and 30 μg/mL of total extract (ext) and HRX-SPE fractions (B–E) in TLR-2 (**A**), TLR-4 (**B**) and Dectin (**C)** NF-kB/SEAP reporter cell lines. Pam2CSK4 (PAM) and LPS were used as positive controls for TLR-2 and TLR-4 respectively, while zymosan was used as positive control on dectin assay. Asterisks indicate significant differences from the cells treated only with vehicle (control, Ctrl) at a 95% ($P < 0.05$) confidence level, as determined using two-way ANOVA analysis.

Figure S2. ^{1}H NMR Bioactive Fraction B (600 MHz, MeOD).

Figure S3. ^{1}H NMR Compound **1** (600 MHz, MeOD).

Figure S4. Mass spectra (ESI positive and negative modes) of compound **1**.

Figure S5. ^{1}H NMR Compound **2** (600 MHz, MeOD).

Figure S6. HSQC NMR Compound **2** (600 MHz, MeOD).

Figure S7. ^{13}C NMR Compound **2** (600 MHz, MeOD).

Figure S8. 1H NMR Compound **2** (600 MHz, DMSO-d6).

Figure S9. HSQC NMR Compound **2** (600 MHz, DMSO-d6).

Figure S10. ^{13}C NMR Compound **2** (600 MHz, DMSO-d6).

Figure S11. Mass spectra (ESI positive and negative modes) of compound **2**.

Figure S12. ^{1}H NMR of fraction containing compounds **3** (600 MHz, MeOD).

Figure S13. ^{1}H-^{1}H COSY spectra of fraction containing compounds **3** (400 MHz, MeOD).

Figure S14. HSCQ NMR spectra of fraction containing compounds **3** (600 MHz, MeOD).

Figure S15. HMBC NMR spectra of fraction containing compounds **3** (600 MHz, MeOD).

Figure S16. 13C NMR spectra of fraction containing compounds 3 (600 MHz, MeOD).

Figure S17. LC-MS/MS (ESI negative mode) of fraction containing compounds **3**.

Figure S18. MS/MS (ESI negative mode) of the main peak at m/z 738.55.

Figure S19. MS/MS (ESI negative mode) of the main peak at m/z 752.57.

Figure S20. MS/MS (ESI negative mode) of the main peak at m/z 780.60.

Figure S21. MS/MS (ESI negative mode) of the main peak at m/z 794.61.

Figure S22. MS/MS (ESI negative mode) of the main peak at m/z 808.63.

Figure S23. MS/MS (ESI negative mode) of the main peak at m/z 822.65.

Figure S 1. Evaluation of the Toll-Like Receptor (TLR) and Dectin 1b activation with 5 and 30 µg/mL of total extract (ext) and HRX-SPE fractions (B–E) in TLR-2 (**A**), TLR-4 (**B**) and Dectin (**C**) NF-kB/SEAP reporter cell lines. Pam2CSK4 (PAM) and LPS were used as positive controls for TLR-2 and TLR-4 respectively, while zymosan was used as positive control on dectin assay. Asterisks indicate significant differences from the cells treated only with vehicle (control, Ctrl) at a 95% ($P < 0.05$) confidence level, as determined using two-way ANOVA analysis.

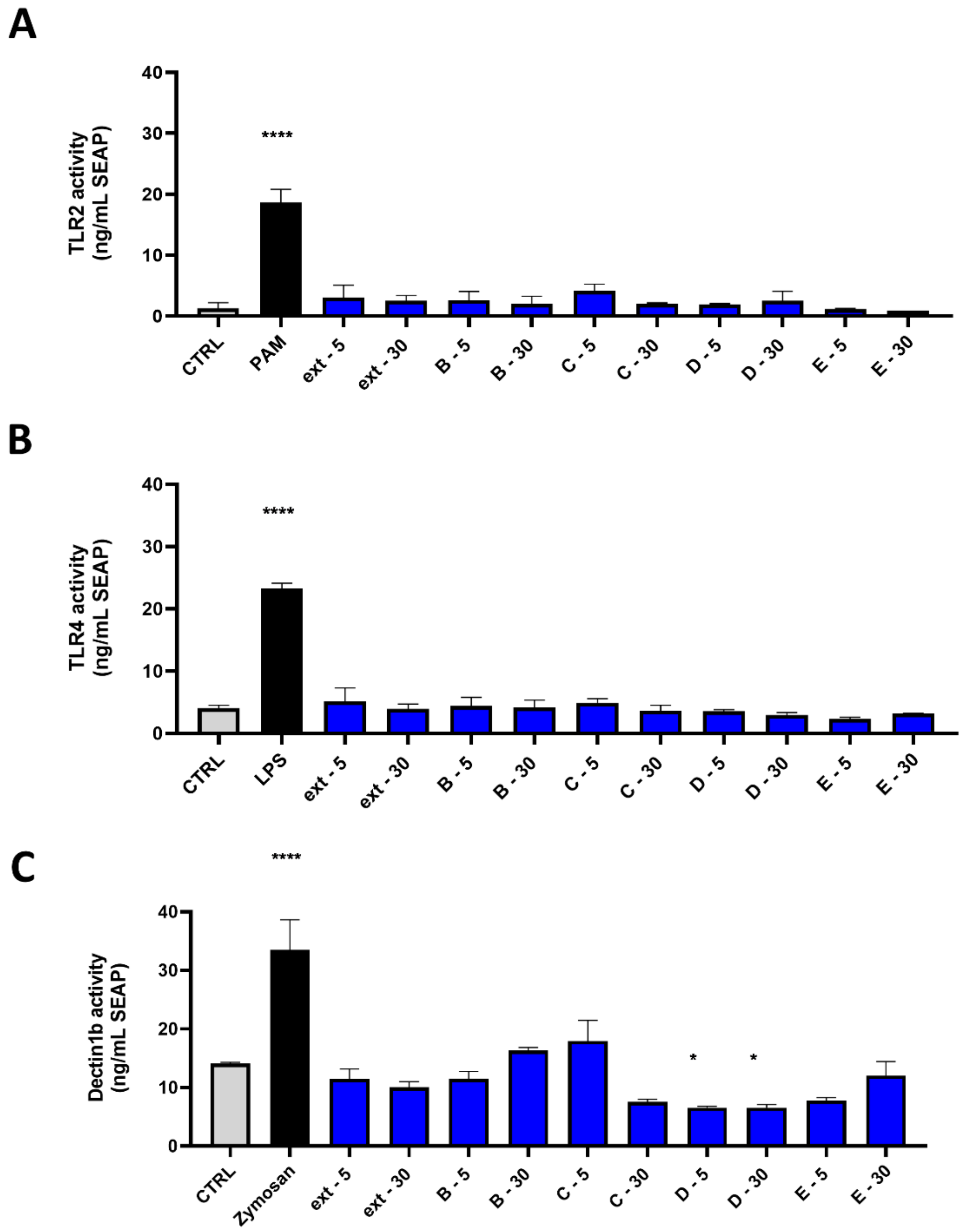

Figure S 2. 1H NMR Bioactive Fraction B (600 MHz, MeOD).

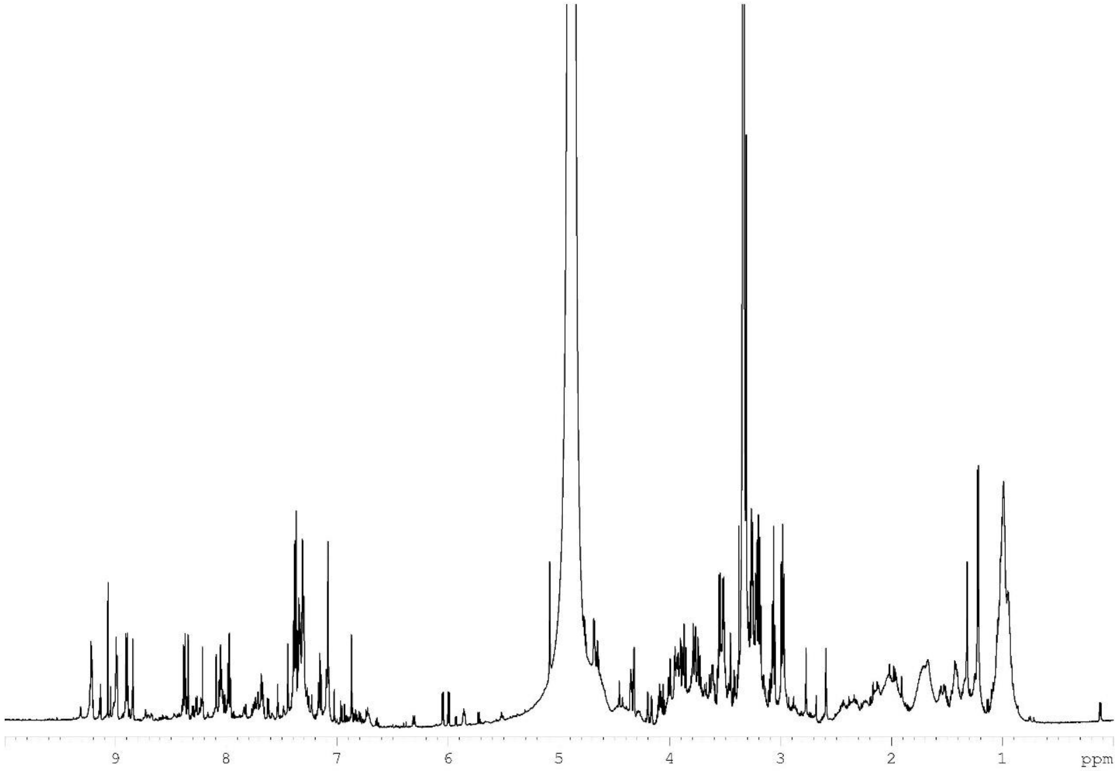

Figure S 3. ^{1}H NMR Compound **1**(600 MHz, MeOD).

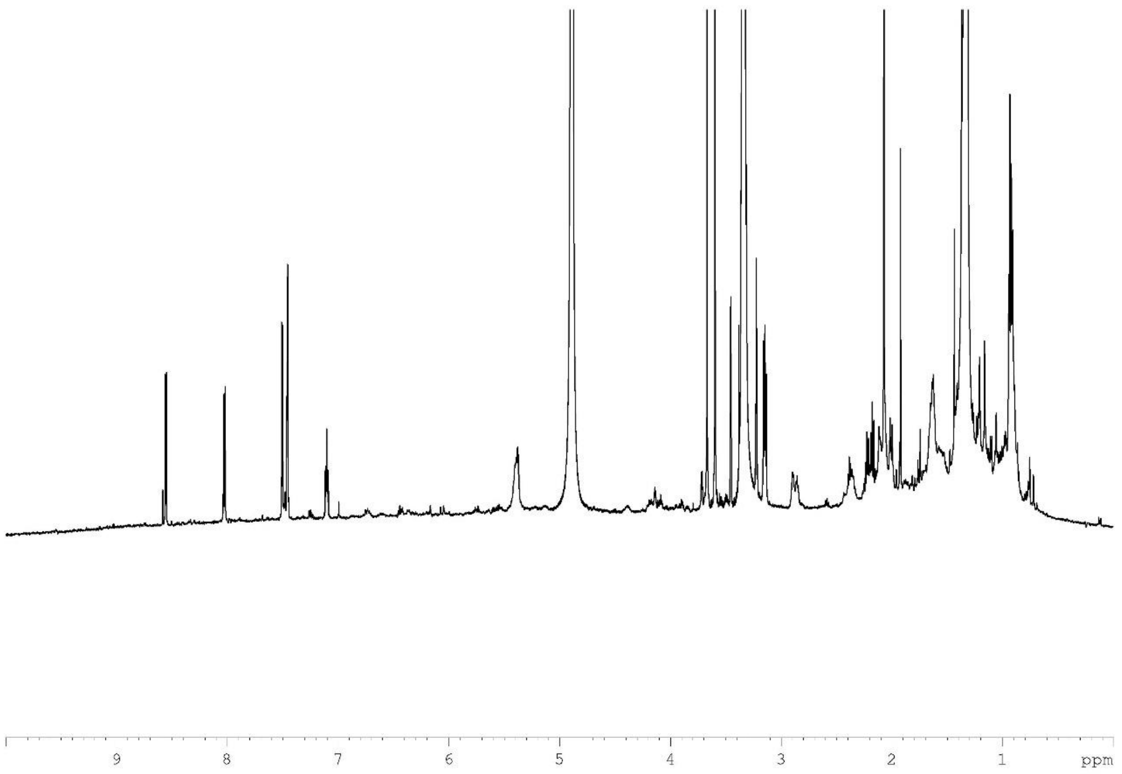

Figure S 4. Mass spectra (ESI positive and negative modes) of compound **1**.

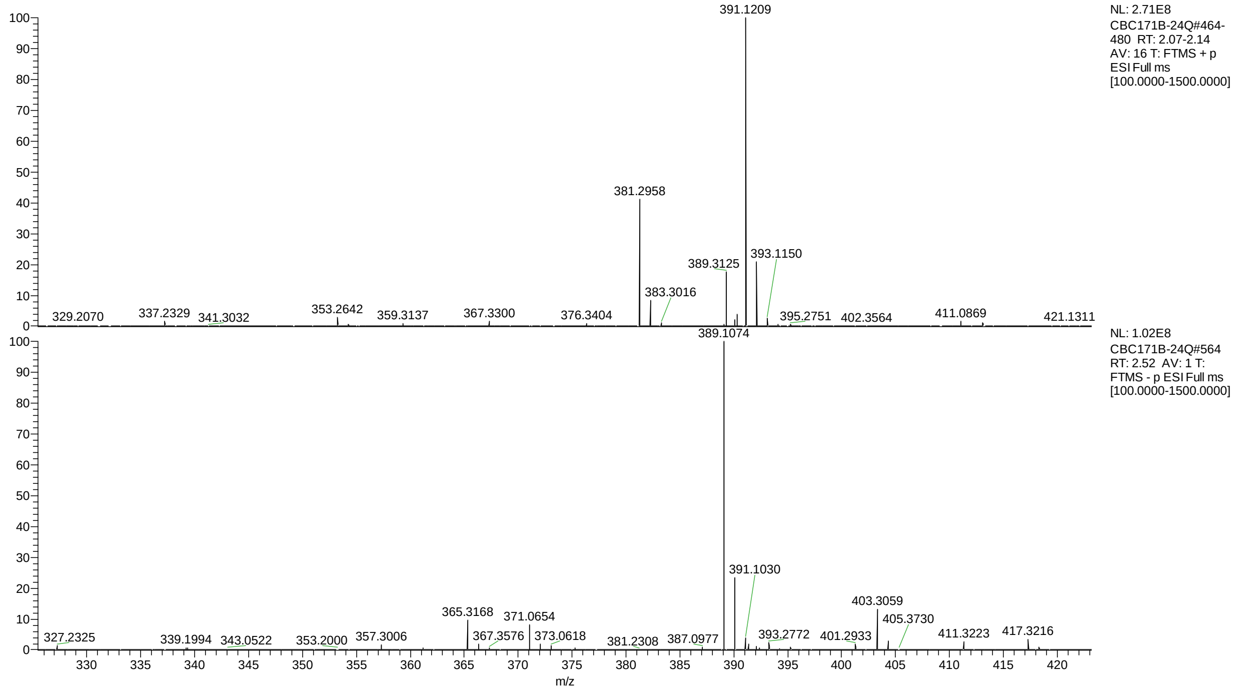

Figure S 5. ^{1}H NMR Compound **2** (600 MHz, MeOD).

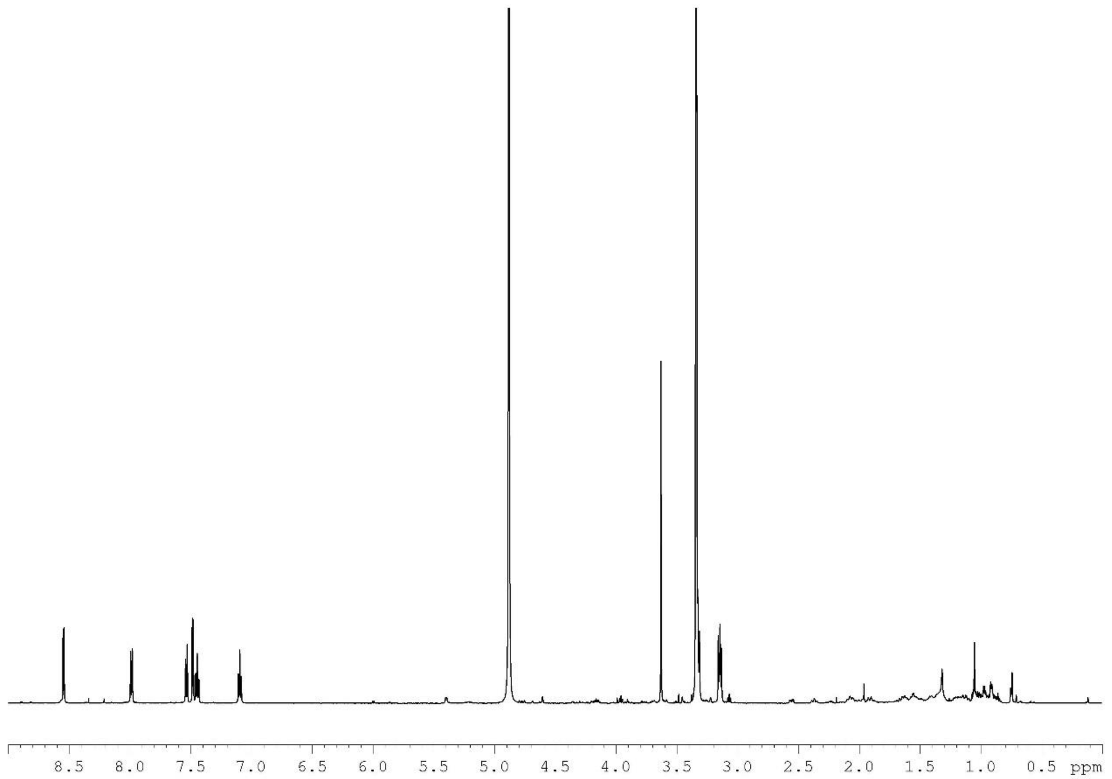

Figure S 6. HSQC NMR Compound **2** (600 MHz, MeOD).

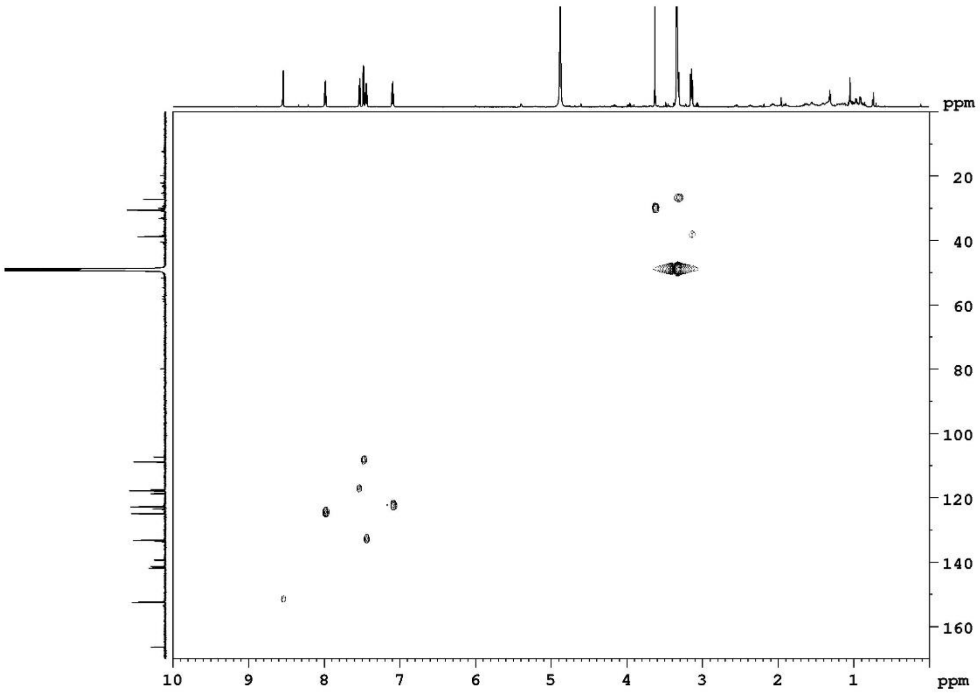

Figure S 7. ^{13}C NMR Compound **2** (600 MHz, MeOD).

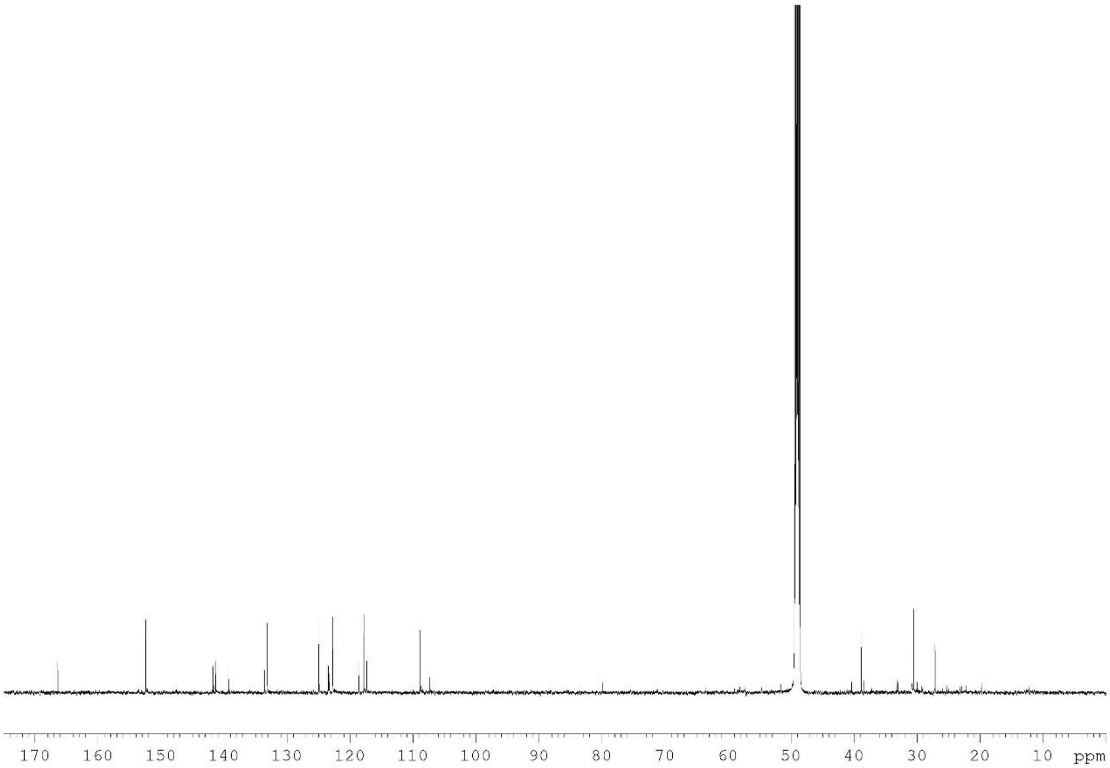

Figure S 8. ^{1}H NMR Compound **2** (600 MHz, DMSO-d_6).

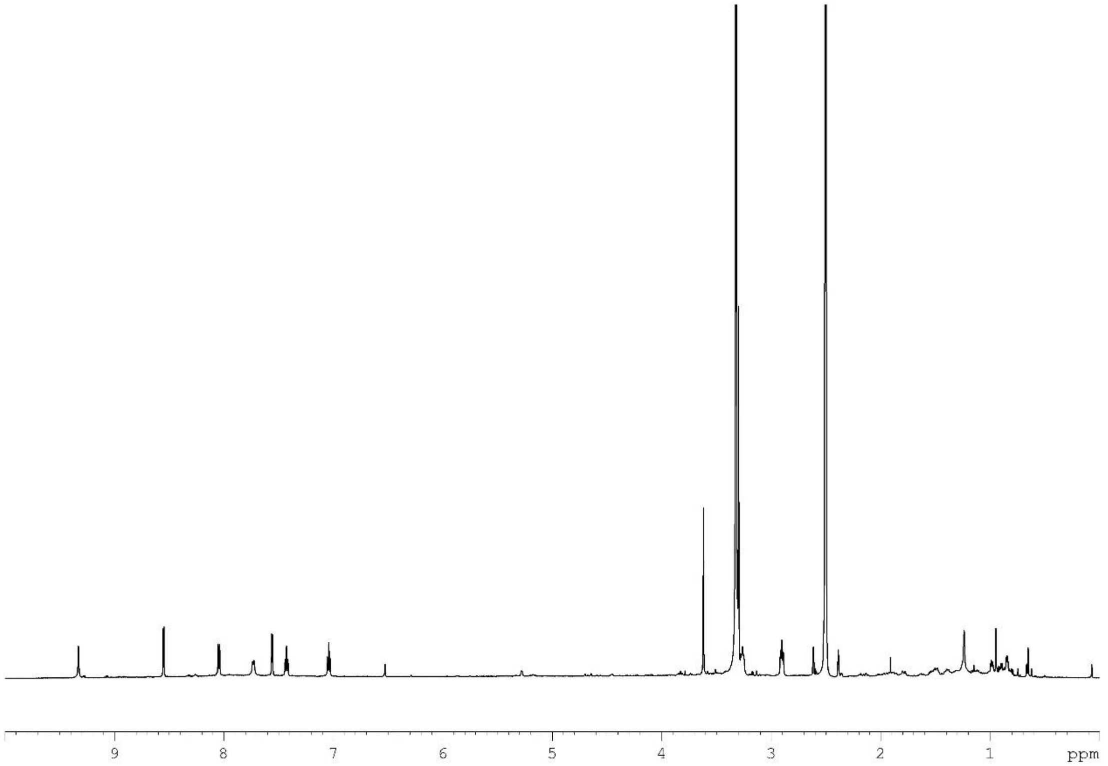

Figure S 9. HSQC NMR Compound **2** (600 MHz, DMSO-d_6).

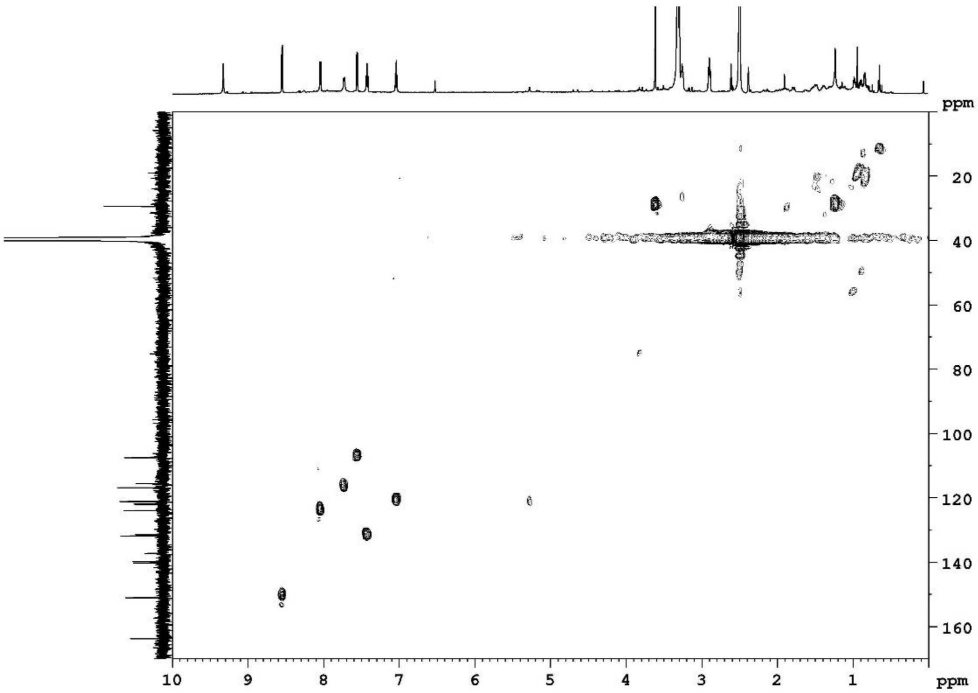

Figure S 10. ^{13}C NMR Compound **2** (600 MHz, DMSO-d_6).

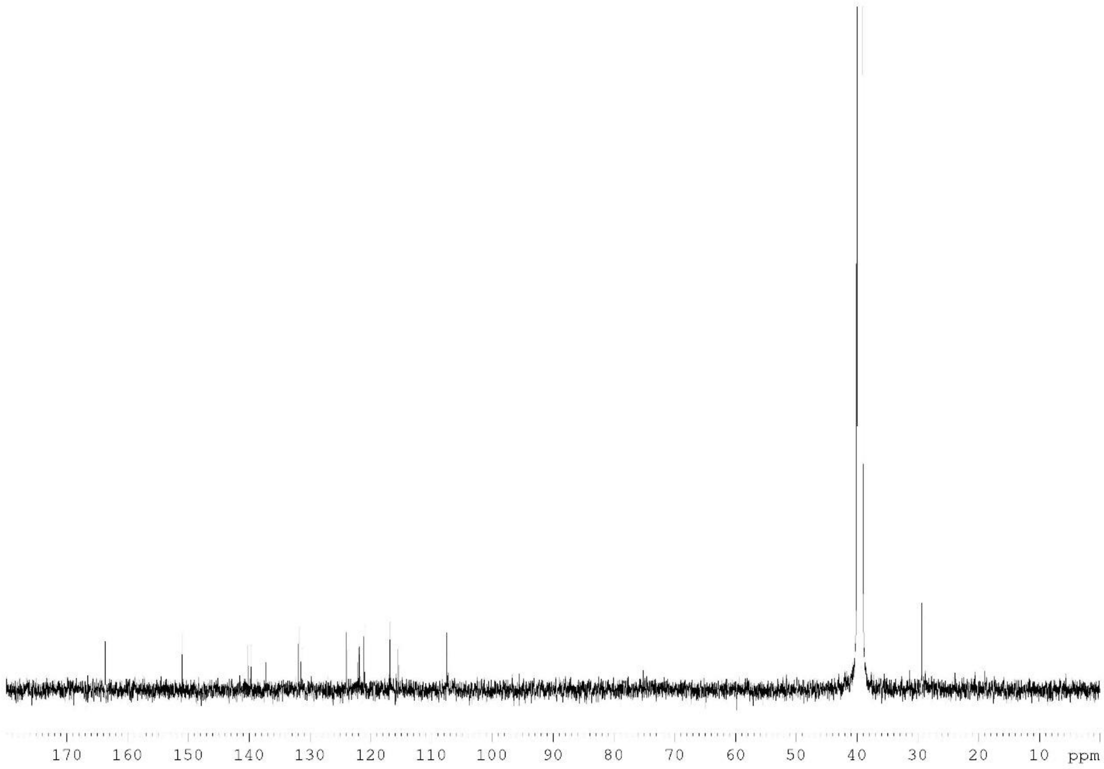

Figure S 11. Mass spectra (ESI positive and negative modes) of compound **2**.

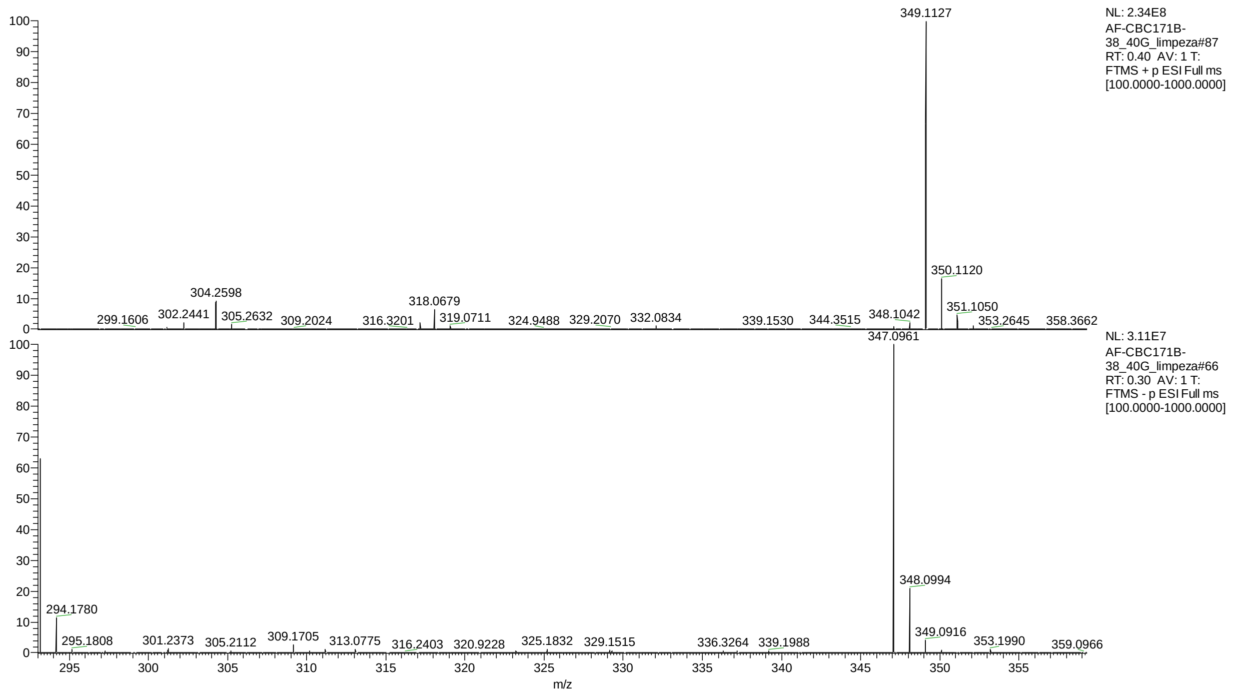

Figure S 12. ^{1}H NMR of fraction containing compounds **3** (600 MHz, MeOD).

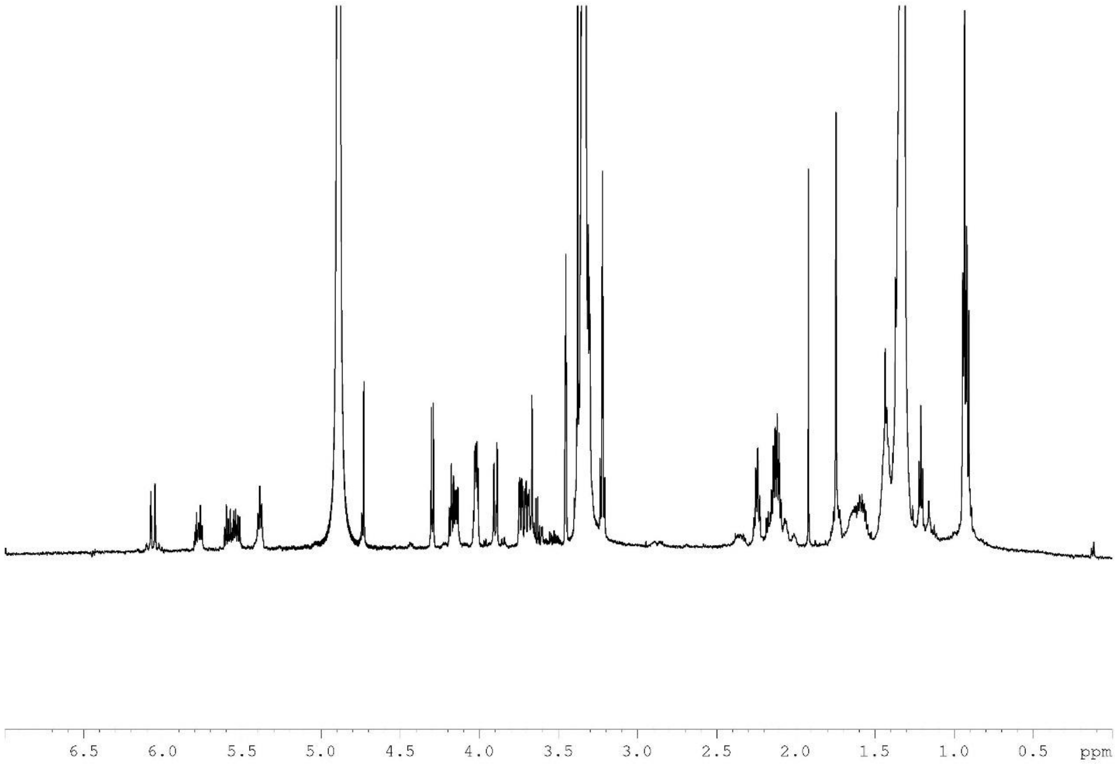

Figure S 13. ^{1}H-^{1}H COSY spectra of fraction containing compounds **3** (400 MHz, MeOD).

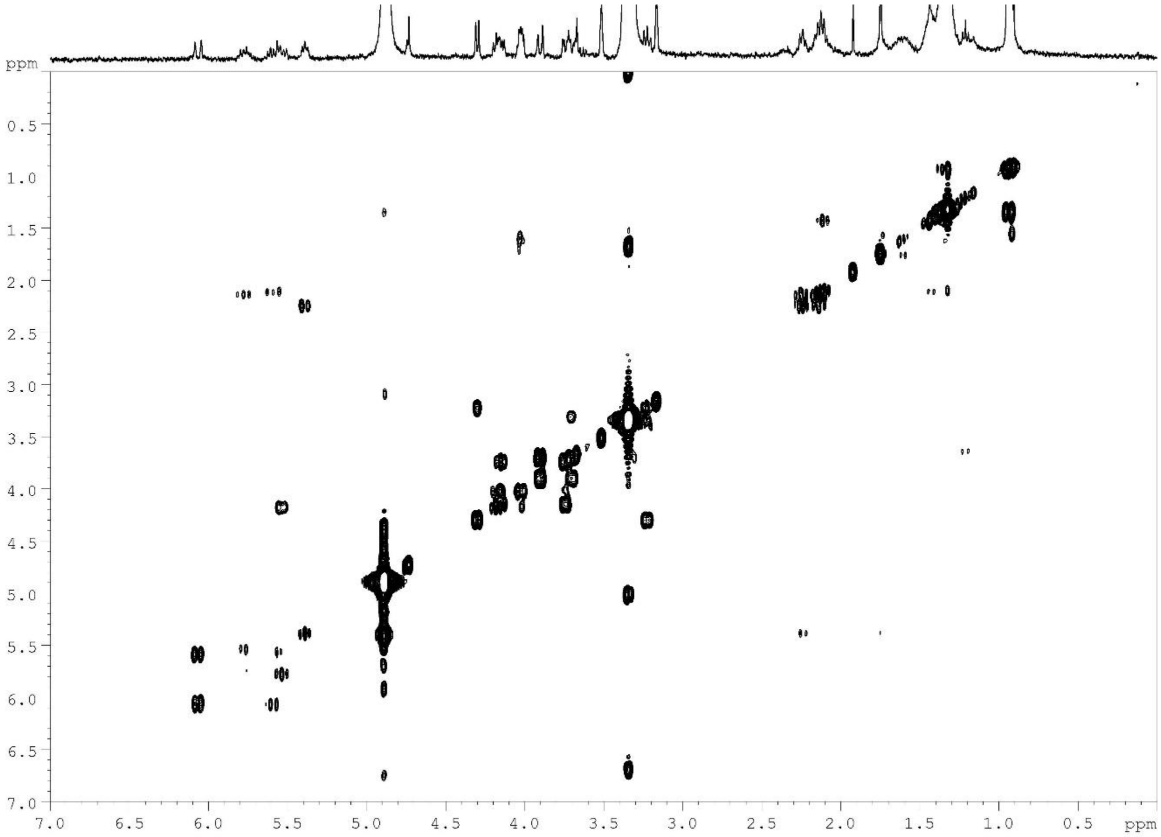

Figure S 14. HSCQ NMR spectra of fraction containing compounds **3** (600 MHz, MeOD).

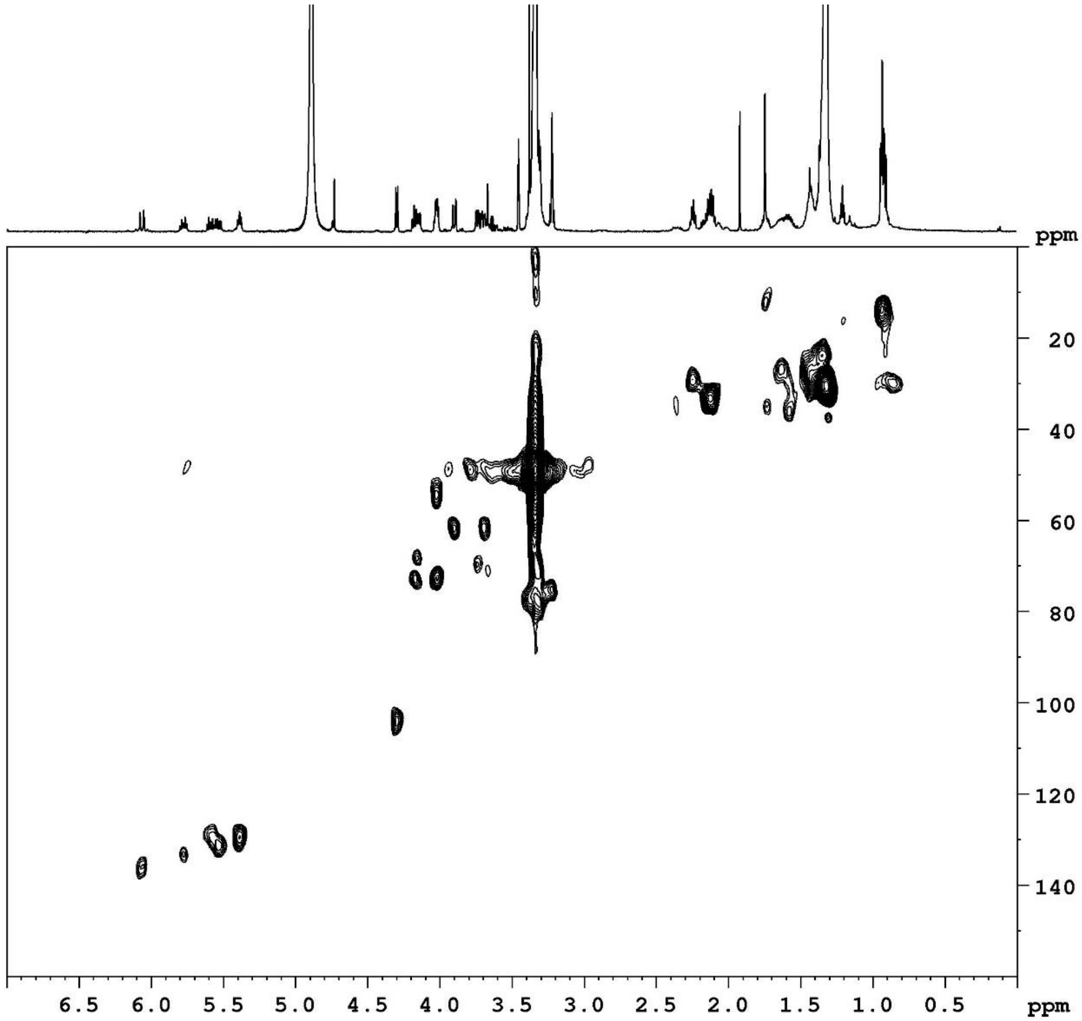

Figure S 15. HMBC NMR spectra of fraction containing compounds **3** (600 MHz, MeOD).

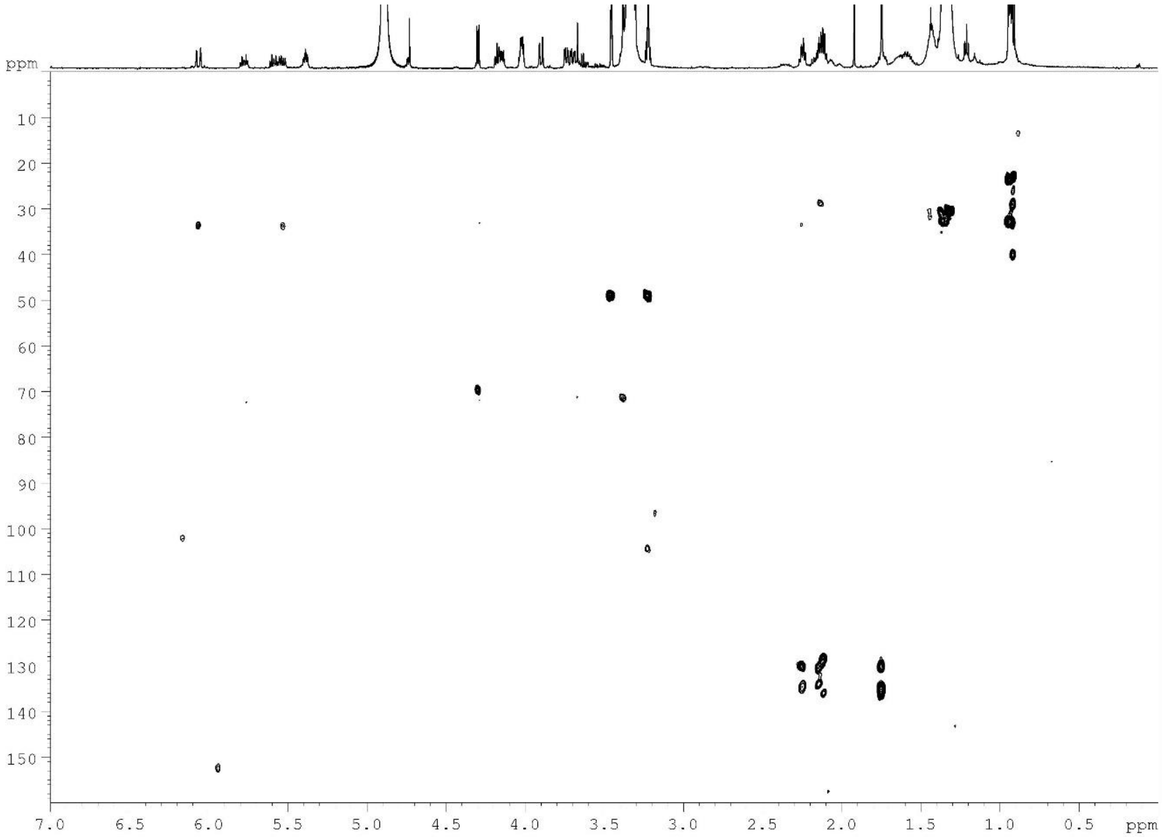

Figure S 16. ^{13}C NMR spectra of fraction containing compounds **3** (600 MHz, MeOD).

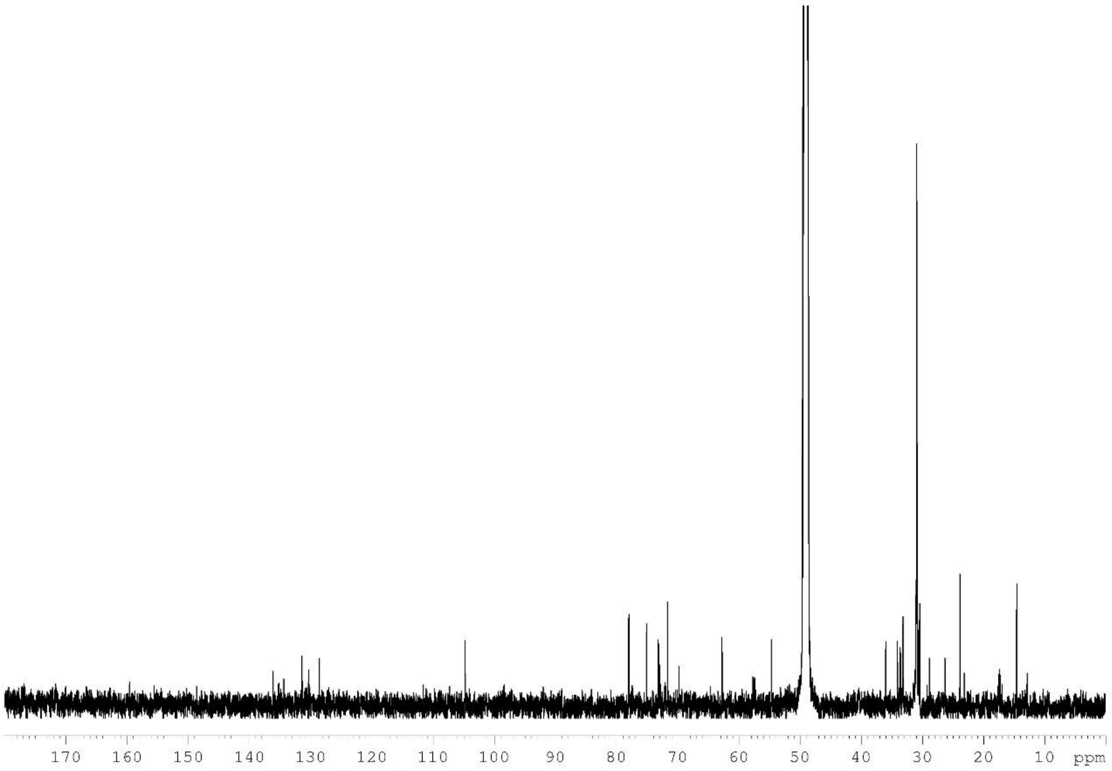

Figure S 17. LC-MS/MS (ESI negative mode) of fraction containing compounds **3**.

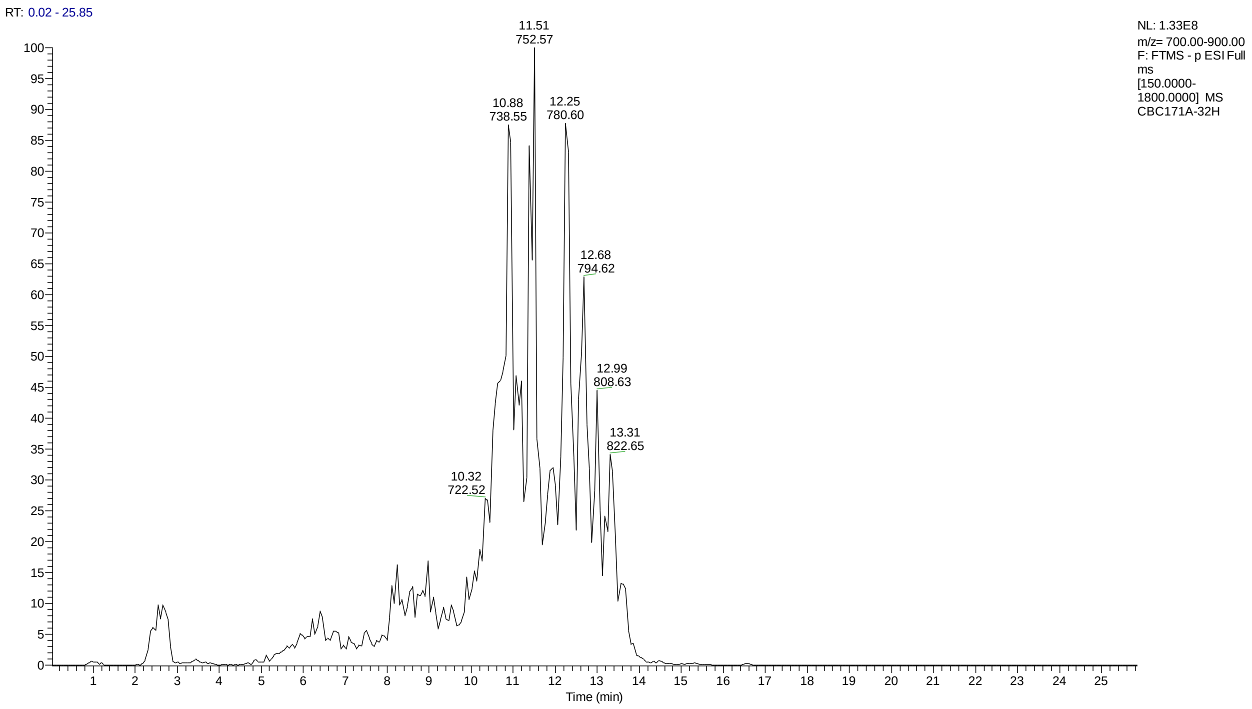

Figure S 18. MS/MS (ESI negative mode) of the main peak at m/z 738.55.

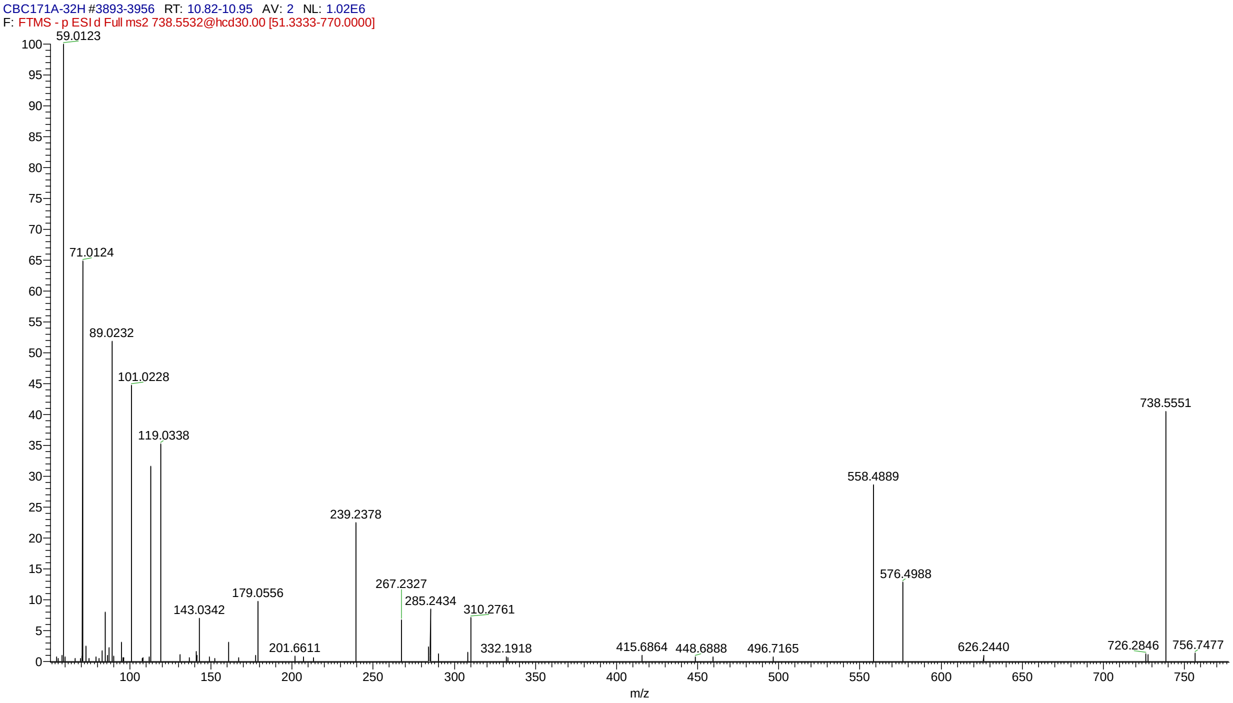

Figure S19. MS/MS (ESI negative mode) of the main peak at m/z 752.57.

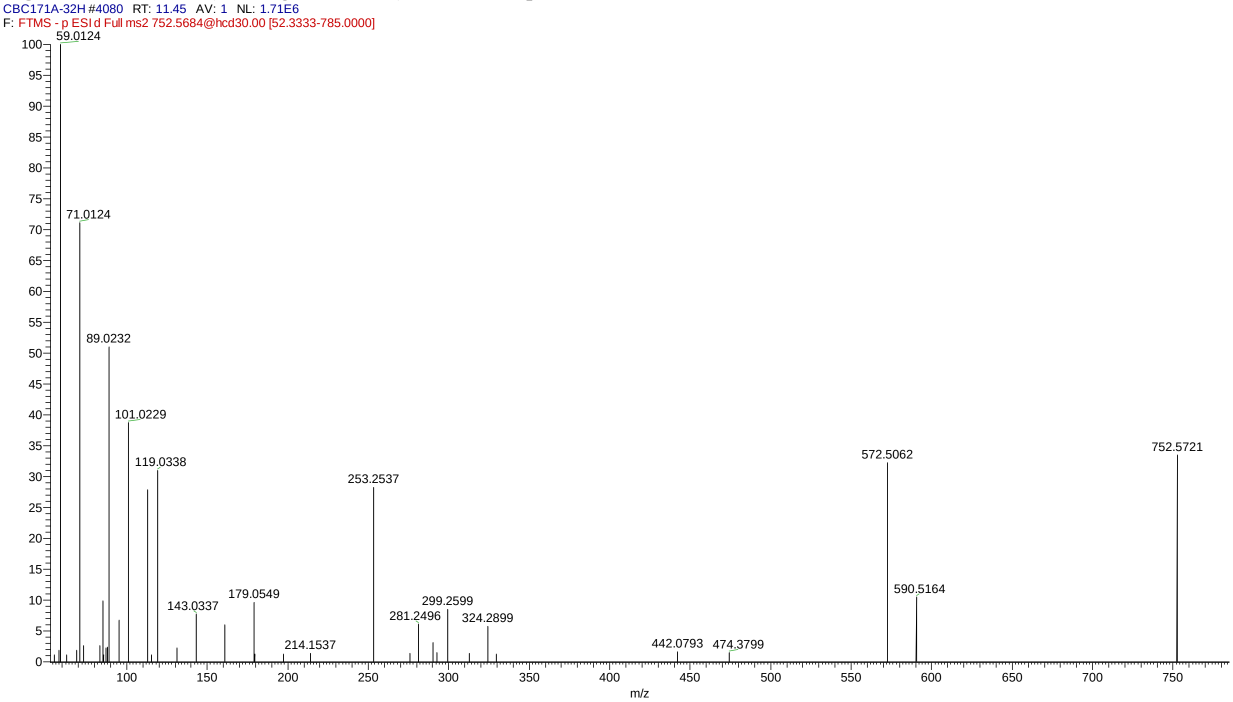

Figure S 20. MS/MS (ESI negative mode) of the main peak at m/z 780.60.

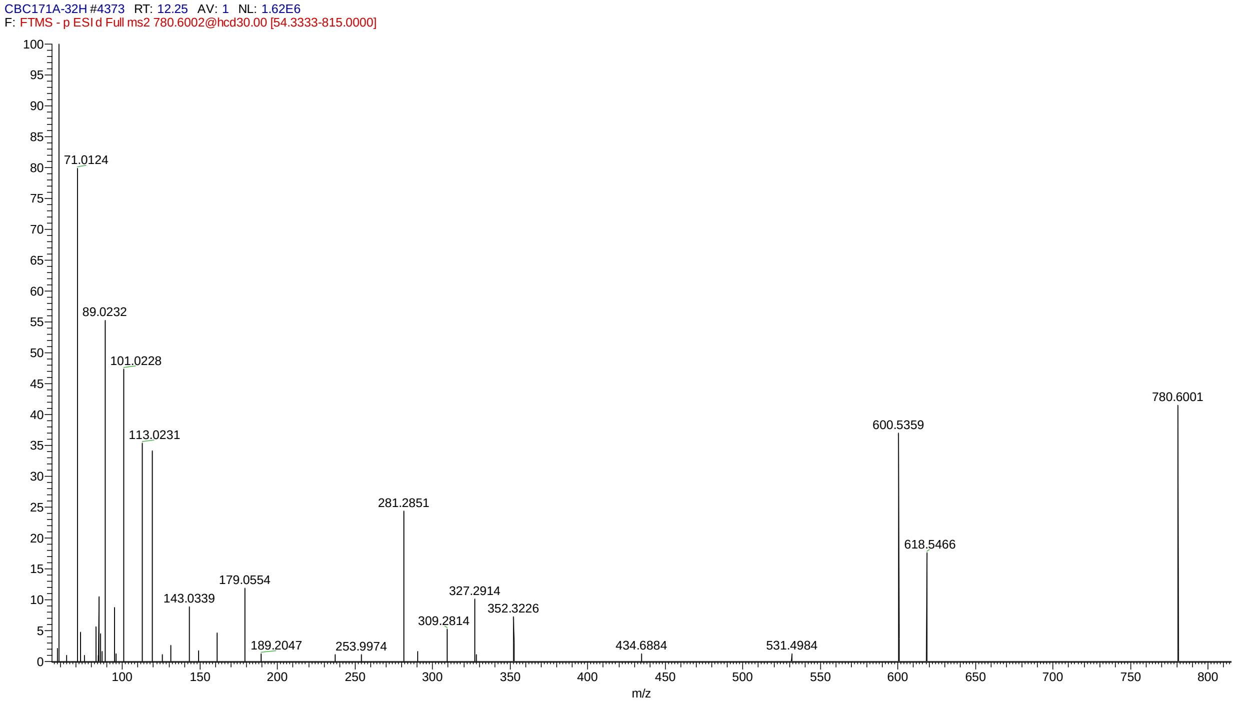

Figure S 21. MS/MS (ESI negative mode) of the main peak at m/z 794.61.

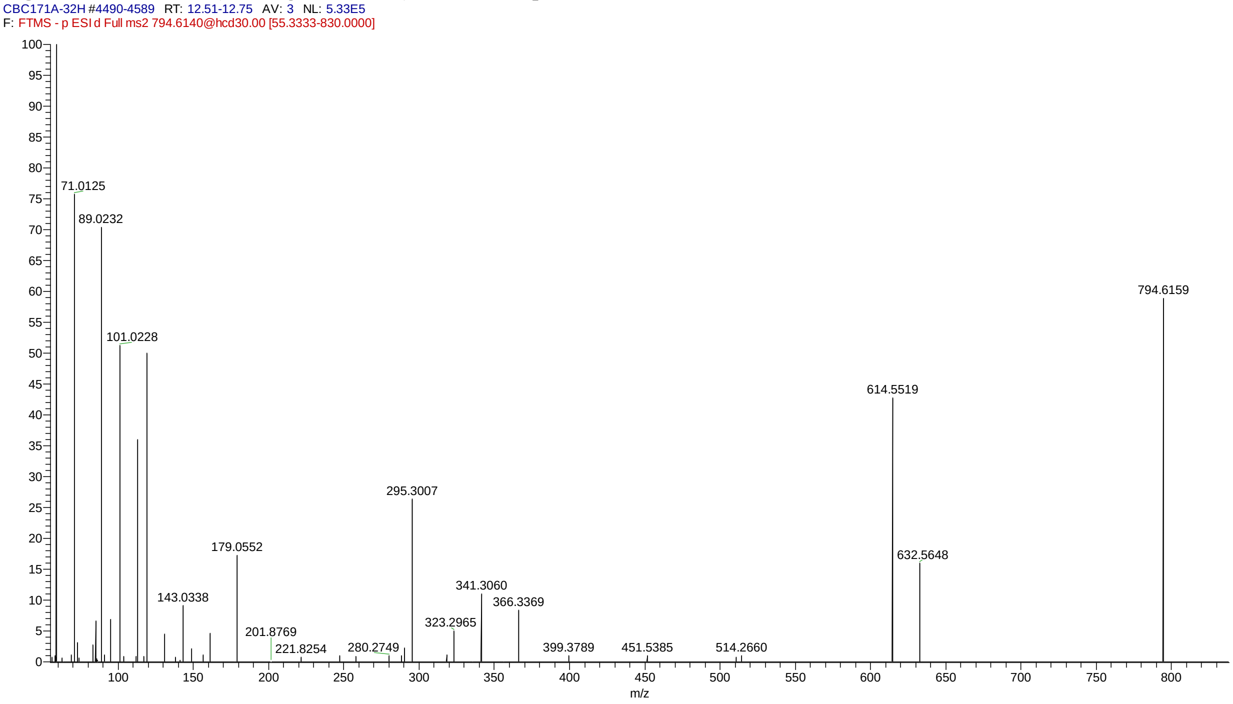

Figure S 22. MS/MS (ESI negative mode) of the main peak at m/z 808.63.

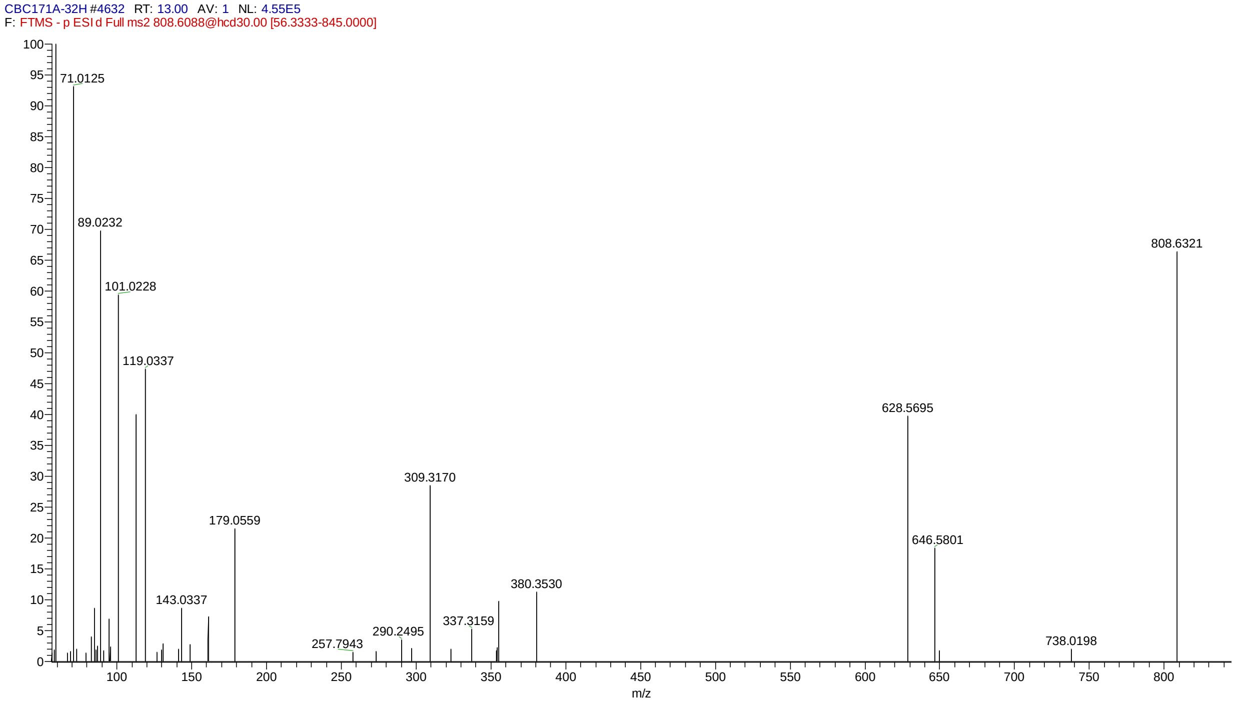

Figure S 23. MS/MS (ESI negative mode) of the main peak at m/z 822.65.

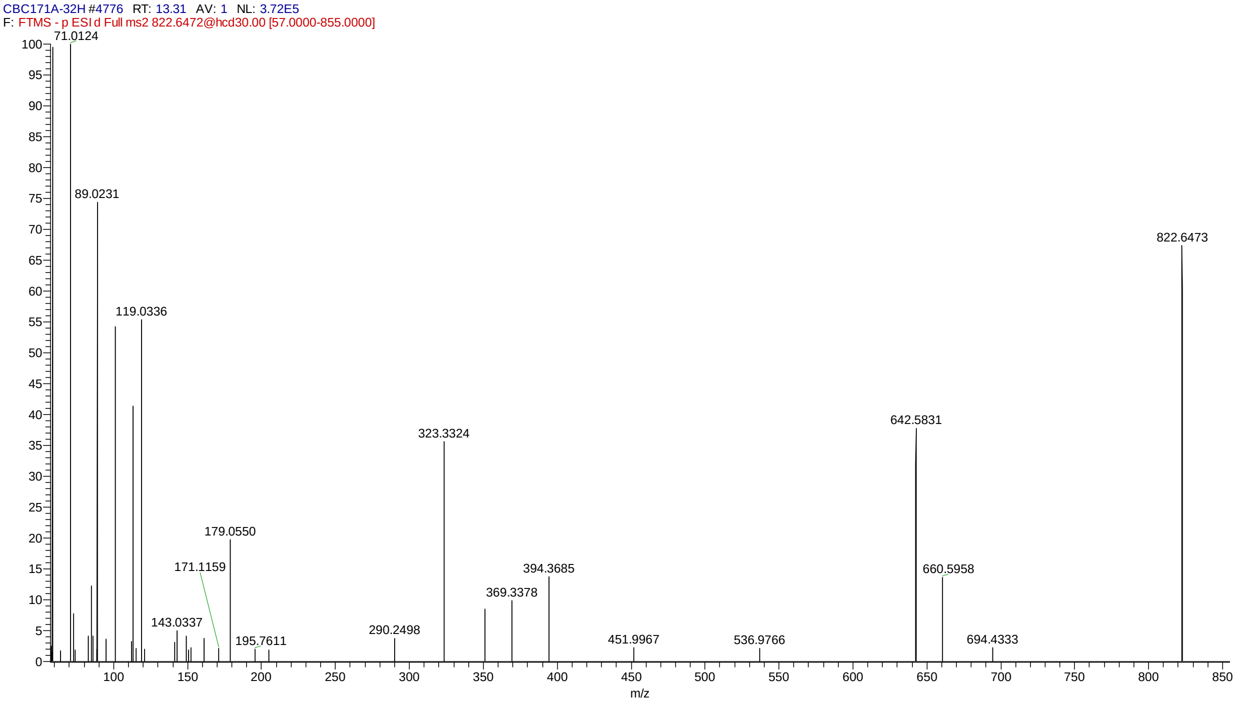

Supplement: Supplementary file 1 [file marinedrugs-22-00075-s001.zip › marinedrugs-2826281-supplementary.pdf]
